# Variations in chloroplast movement and chlorophyll fluorescence among chloroplast division mutants under light stress

**DOI:** 10.1093/jxb/erx203

**Published:** 2017-06-22

**Authors:** Siddhartha Dutta, Jeffrey A Cruz, Saif M Imran, Jin Chen, David M Kramer, Katherine W Osteryoung

**Affiliations:** 1 Department of Plant Biology, Michigan State University, East Lansing, MI, USA; 2 MSU-DOE-Plant Research Laboratory, Michigan State University, East Lansing, MI, USA; 3 Department of Biochemistry and Molecular Biology Michigan State University, East Lansing, MI, USA; 4 Department of Electrical and Computer Engineering, Michigan State University, East Lansing, MI, USA; 5 Department of Computer Sciences and Engineering, Michigan State University, East Lansing, MI, USA

**Keywords:** Chloroplast division mutants, chlorophyll fluorescence, chloroplast movement, chloroplast size, light stress, photosynthesis

## Abstract

Chloroplasts divide to maintain consistent size, shape, and number in leaf mesophyll cells. Altered expression of chloroplast division proteins in Arabidopsis results in abnormal chloroplast morphology. To better understand the influence of chloroplast morphology on chloroplast movement and photosynthesis, we compared the chloroplast photorelocation and photosynthetic responses of a series of Arabidopsis chloroplast division mutants with a wide variety of chloroplast phenotypes. Chloroplast movement was monitored by red light reflectance imaging of whole plants under increasing intensities of white light. The accumulation and avoidance responses were differentially affected in different mutants and depended on both chloroplast number and morphological heterogeneity. Chlorophyll fluorescence measurements during 5 d light experiments demonstrated that mutants with large-chloroplast phenotypes generally exhibited greater PSII photodamage than those with intermediate phenotypes. No abnormalities in photorelocation efficiency or photosynthetic capacity were observed in plants with small-chloroplast phenotypes. Simultaneous measurement of chloroplast movement and chlorophyll fluorescence indicated that the energy-dependent (qE) and long-lived components of non-photochemical quenching that reflect photoinhibition are affected differentially in different division mutants exposed to high or fluctuating light intensities. We conclude that chloroplast division mutants with abnormal chloroplast morphologies differ markedly from the wild type in their light adaptation capabilities, which may decrease their relative fitness in nature.

## Teaser

Chloroplast division mutants with enlarged chloroplasts or heterogeneous chloroplast populations differ markedly from the wild type in their photorelocation efficiency and/or photosynthetic capacity under high or fluctuating light intensities.

## Introduction

Chloroplasts are highly dynamic organelles that continuously regulate their size, shape, and numbers (Pyke, 2013). These dynamic processes play a critical role in cell physiology. Apart from being responsible for photosynthesis, chloroplasts provide a multifunction platform to the plant cell, contributing to the synthesis of lipids, amino acids, nucleotides, and various hormones, and to nitrogen and sulphur assimilation (Ohlrogge and Browse, 1995; Neuhaus and Emes, 2000; Lopez-Juez and Pyke, 2005). Because of their semi-autonomous nature (Timmis *et al.*, 2004), all of these diverse functions are tightly regulated by both interchloroplastic crosstalk and communication with other cell organelles (Raghavendra and Padmasree, 2003; Nott *et al.*, 2006; Jarvis and Lopez-Juez, 2013; Bulychev and Komarova, 2015). Chloroplast continuity during cell division and their accumulation to high numbers in photosynthetic tissues are maintained by division of pre-existing chloroplasts (Osteryoung and Pyke, 2014).

Chloroplast division in Arabidopsis is mediated by mid-plastid-localized stromal and cytosolic contractile complexes, respectively designated the filamenting temperature-sensitive Z (FtsZ) ring and Accumulation and Replication of Chloroplasts 5 (ARC5)/Dynamin-Related Protein 5B (DRP5B) ring (Vitha *et al.*, 2001; Gao *et al.*, 2003; Hong *et al.*, 2003). The FtsZ ring, consisting of the cytoskeletal GTPase proteins FtsZ1 and FtsZ2, is anchored to the stromal face of the inner envelope membrane by ARC6 (Pyke and Leech, 1994; Vitha *et al.*, 2001, 2003; Olson *et al.*, 2010; Smith *et al.*, 2010; TerBush and Osteryoung, 2012; Yoshida *et al*, 2016). The midcell positioning of the FtsZ ring is regulated by a complex regulatory ‘Min’ system, comprising MinD1, MinE1, ARC3, and MULTIPLE CHLOROPLAST DIVISION SITE 1 (MCD1) (Colletti *et al.*, 2000; Itoh *et al.*, 2001; Maple *et al.*, 2002; Maple and Møller, 2007; Nakanishi *et al.*, 2009; Zhang *et al.*, 2013). The ARC6-like protein PARALOG of ARC6 (PARC6) also aids in FtsZ ring assembly and positioning (Maple *et al.*, 2007; Glynn *et al.*, 2009; Zhang *et al.*, 2016). The ARC5/DRB5P ring is recruited to the division site by the outer envelope proteins PLASTID DIVISION1 (PDV1) and PLASTID DIVISION2 (PDV2) (Miyagishima *et al.*, 2006; Holtsmark *et al.*, 2013). Mutations in and/or overexpression of components of the chloroplast division machinery alter chloroplast size and number in Arabidopsis, yielding cells with abnormal chloroplast morphologies depending on the genotype (Table 1). Most mutations result in fewer, larger chloroplasts than in the wild type (WT) (Osteryoung and Pyke, 2014), but overexpression of PDV1 and PDV2 produces plants with smaller and more numerous chloroplasts (Okazaki *et al.*, 2009). Although the chloroplast protein FZL is not part of the division machinery, *fzl* knockout mutants also have altered chloroplast morphology phenotypes (Gao *et al.*, 2006) (Table 1).

**Table 1.** Properties of Arabidopsis genotypes with abnormal chloroplast morphologies used for this study

Genotype	Locus	Parental line	Chloroplast phenotype	Reference
Large chloroplasts				
*arc5-2*	At3g19720	Col-0	3–6 giant chloroplasts, centrally constricted	Miyagishima *et al.* (2006)
*arc6-5*	At5g42480	Col-0	1–2 giant chloroplasts	Crumpton-Taylor *et al.* (2012)
*arc12*	At1g69390	Col-0	1–2 giant chloroplasts	Glynn *et al.* (2007)
*pdv1-1*	At5g53280	Col-0	2–5 giant chloroplasts, with constrictions	Miyagishima *et al.* (2006)
*pdv2-1*	At2g16070	Col-0	3–6 giant chloroplasts, with constrictions	Miyagishima *et al.* (2006)
*pdv1-1 pdv2-1*	At5g53280, At2g16070	Col-0	1–2 giant chloroplasts, with central constriction	Miyagishima *et al.* (2006)
Intermediate chloroplasts				
*ftsZ1-1*	At5g55280	Col-0	Heterogenous, enlarged chloroplasts with some small chloroplasts	Yoder *et al.* (2007)
*ftsZ2-2*	At3g52750	Col-0	Fewer, slightly enlarged, uniform size	McAndrew *et al.* (2008)
*arc3-2*	At1g75010	Col-0	~11 irregularly globular, large chloroplasts	Shimada *et al.* (2004)
*parc6-1*	At3g19180	Col-0	~7 heterogenous, irregular chloroplasts, some with constrictions	Glynn *et al.* (2009)
*arc11/minD1*	At5g24020	L*er*	Heterogenous, giant chloroplasts with some small chloroplasts	Marrison *et al.* (1999)
*minD1-1*	At5g24020	Ws	Heterogenous, giant chloroplasts with some small chloroplasts	Zhang *et al.* (2013)
*fzl*	At1g03160	Col-0	Fewer, larger chloroplasts, heterogenous in distribution	Gao *et al.* (2006)
Small chloroplasts				
*35S PDV1 35S -PDV2*	At5g53280, At2g16070	Col-0	More, smaller chloroplasts	Okazaki *et al.* (2009)

Despite the change in chloroplast size and number due to altered expression of division genes in Arabidopsis, the total chloroplast compartment area per unit of cell area in mesophyll cells was found to be equal to that in the WT (Pyke and Leech, 1992, 1994; Osteryoung *et al.*, 1998). Additionally, mutants with varying chloroplast morphologies and numbers are viable and grow normally under controlled environmental conditions (Supplementary Fig. S1 at *JXB* online) (Pyke and Leech, 1994; Marrison *et al.*, 1999; Shimada *et al.*, 2004; Gao *et al.*, 2006; Miyagishima *et al.*, 2006; Glynn *et al.*, 2007, 2008, 2009; Yoder *et al.*, 2007; McAndrew *et al.*, 2008; Zhang *et al.*, 2013). Thylakoid organization is also largely normal in chloroplast division mutants, though some anomalies are observed (Pyke *et al.*, 1994; Marrison *et al.*, 1999; Shimada *et al.*, 2004; Austin and Webber, 2005; Gao *et al.*, 2006).

Reduced photosynthetic efficiency has been reported in severe chloroplast division mutants of Arabidopsis and in transgenic *Nicotiana tabacum* plants overexpressing an *FtsZ* gene (Jeong *et al.*, 2002; Königer *et al.*, 2008). These plants have enlarged chloroplasts that exhibit impaired photorelocation responses under low and high light regimes. Chloroplast photorelocation (movement) is an adaptive mechanism by which chloroplasts are repositioned within mesophyll cells to optimize light absorption in low light (accumulation response, in which chloroplasts move to the periclinal walls, adopting the ‘face position’) and minimize photodamage in high light (avoidance response, in which chloroplast align along the anticlinal walls, adopting the ‘profile position’) (Senn, 1908; Haupt, 1973; Wada *et al.*, 1993; Trojan and Gabryś, 1996; Augustynowicz and Gabryś, 1999; Kasahara *et al.*, 2002; Sztatelman *et al.*, 2010; Davis and Hangarter, 2012; Wada, 2013). The photosynthetic phenotypes measured by Jeong *et al.* (2002) and Königer *et al.* (2008) were attributed largely to inefficient photorelocation by the enlarged chloroplasts, although the photosynthetic defects in a few large chloroplast mutants were also ascribed to altered composition and structure of the photosynthetic apparatus (Austin and Webber, 2005).

We recently reinvestigated the photorelocation and photosynthetic responses of three severe chloroplast division mutants with only 1–2 drastically enlarged chloroplasts in their mesophyll cells using a non-invasive, whole-plant imaging approach (Dutta *et al.*, 2015). Somewhat surprisingly, we found that the photosynthetic phenotypes of these mutants were attributable largely to altered chloroplast size and shape rather than to diminished chloroplast movement capacity (Dutta *et al.*, 2015). Our imaging platform also allowed us to tease apart the contributions of PSII quantum efficiency (Φ_II_) and non-photochemical quenching (NPQ) to the high-light susceptibility of these mutants. In the present study, we have extended our analysis to include a series of Arabidopsis genotypes that exhibit a wider array of chloroplast morphology phenotypes.

## Materials and methods

### Plant materials and growth conditions

*Arabidopsis thaliana* lines used in this study (Table 1) include: T-DNA insertion mutants *arc5-2* (Miyagishima *et al.*, 2006), *arc6-5* (Crumpton-Taylor *et al.*, 2012), *pdv2-1* (Miyagishima *et al.*, 2006), *ftsZ1-1* (Yoder *et al.*, 2007), *ftsZ2-2* (McAndrew *et al.*, 2008), *arc3-2* (Shimada *et al.*, 2004), *parc6-1* (Glynn *et al.*, 2009), and *fzl* (Gao *et al.*, 2006) in the Col-0 background; the T-DNA insertion mutant *minD1-1* in the Ws background (Zhang *et al.*, 2013); ethyl methanesulfonate (EMS) mutants *arc12* (Glynn *et al.*, 2007), *pdv1-1*, and *pdv1-1 pdv2-1* (Miyagishima *et al.*, 2006) in the Col-0 background; the EMS mutant *arc11* in the L*er* background (Marrison *et al.*, 1999); and a line overexpressing PDV1 and PDV2 in Col-0 (*35S-PDV1 35S-PDV2*) (Okazaki *et al.*, 2009).

Seeds were sown on soil in individual pots and stratified at 4 °C for 48 h in the dark. Plants were germinated and grown in controlled-environment chambers at 20 °C and 60% humidity with a 16/8 h light/dark cycle in white light at 100 µmol photons m^–2^ s^–1^. Plants were transferred to the imaging chamber (photoperiod synchronized to the growth chamber) 1–2 d before an experiment for acclimation.

### Confocal microscopy

For analysis of chloroplast arrangement, entire rosette leaves from 3-week-old plants were fixed and analyzed as described previously (Pyke and Leech, 1991; Dutta *et al.*, 2015). Whole leaf samples were mounted, and mesophyll cells on the adaxial side of leaves were observed using an Olympus Fluoview 1000 Confocal microscope (Olympus Corporation of the Americas Inc., http://www.olympusamerica.com) excited with a 514 nm laser. Chlorophyll (Chl) was detected using a 650 nm long-pass filter. Image analysis was performed using ImageJ version 1.47 (National Institute of Health, http://www.nih.gov).

### Chlorophyll fluorescence measurements

Chl fluorescence imaging of intact plants was performed in a Percival AR41L2 chamber (Geneva Scientific, Fontana, WI, USA) refitted as a dynamic environment photosynthesis imager (DEPI) (Cruz *et al.*, 2016). The initial fluorescence, *F*_0_, was recorded by turning on a weak measuring light. Then, the plants were exposed to a 0.3 s saturation flash of ~10 000 µmol m^–2^ s^–1^, to obtain the maximal fluorescence, *F*_M_. The images were processed using software described in Cruz *et al.* (2016). The quantum yield of PSII (Φ_II_) was calculated as (F_M_'–*F*_S_)/*F*_M_', where *F*_S_ is the steady-state fluorescence and *F*_M_' is the fluorescence maximum at steady state (Baker, 2008). NPQ was estimated using the equation (*F*_M_–*F*_M_')/*F*_M_' (Baker and Oxborough, 2004). The components of NPQ, specifically energy-dependent quenching (qE_SV_) and ‘irreversible’ quenching (qI), were calculated as *F*_M_/*F*_M_'–*F*_M_/*F*_M_'' and (*F*_M_–*F*_M_'')/*F*_M_'', respectively, where *F*_M_'' is the post-illumination fluorescence maximum (Krause and Jahns, 2003). Heat maps were generated with OLIVER software (Dutta *et al.*, 2015).

### Chloroplast relocation assay using red light reflectance imaging

Chloroplast movement was measured by monitoring white light-dependent changes in red light reflectance from whole plants as described (Dutta *et al.*, 2015). Briefly, a DEPI prototype with a CCD camera (KP-FD145GV monochrome, Hitachi Kokusai Electric Inc., Tokyo, Japan) fitted with a 650BP100 band-pass filter (Omega Optical Inc., Brattleboro, VT, USA) was used to collect reflected red light (625 nm) from LEDs also used to excite Chl *a* fluorescence. For each measurement, a 50 μs pulse of the red measuring light was triggered and 30 images were collected over 1.8 s (16.7 Hz) for averaging. Reflectance images were processed using ImageJ (Schneider *et al.*, 2012). Reflectance from whole plants was quantified as described previously (Dutta *et al.*, 2015). Relative reflectance was calculated as the difference between reflectance during illumination (*R*) and the last reflectance value recorded during an initial dark period (*R*_0_), normalized to *R*_0_. Data were analyzed and visualized using software described in Cruz *et al.* (2016) and the Origin visualization package (OriginLab, Northampton, MA, USA).

### Dual imaging and analysis for non-photochemical quenching

Simultaneous imaging of chloroplast movement and fluorescence was performed in the DEPI refitted with a second camera configured to collect reflectance. NPQ values were corrected for interference from chloroplast movement (NPQ_corr_) using the protocol described in Dutta *et al.* (2015), with the following equations: NPQ_corr_= $c\prime \frac{F_{M}}{F_{M}^{\prime }}-1$ , qE_SVcorr_= $c\prime \frac{F_{M}}{F_{M}^{\prime }}-c\prime \prime \frac{F_{M}}{F_{M}^{\prime \prime }}$ , and qI_corr_= $c\prime \prime \;\frac{F_{M}}{F_{M}^{\prime \prime \;}}-1,$ where *c*′ and *c*″ are correction factors to account for changes in light reception caused by chloroplast movements. The values of *c*′ and *c*″ were estimated from reflectance images using the equations: $c\prime =1+m\frac{R^{\prime }-R_{0}}{R_{0}}$ and $c\prime \prime =1+m\frac{R^{\prime \prime }-R_{0}}{R_{0}}$ , where *R*_0_ is the reflectance at time zero, *R*' and *R*'' are the reflectance values measured at the times *F*_M_' and *F*_M_'' were taken, and *m* is the slope of the relationship between fluorescence yield and changes in reflectance. As reported previously (Dutta *et al.*, 2015), the value of *m* for Arabidopsis was determined empirically to be ~1.

### Pigment estimation

Chl and carotenoid contents in leaves of untreated or high-light-treated plants were estimated from *N*,*N*′-dimethylformamide leaf extracts as described by Porra (2002) and Wellburn (1994), respectively.

## Results

### Characterization of chloroplast photorelocation responses

**Fig. 1.**
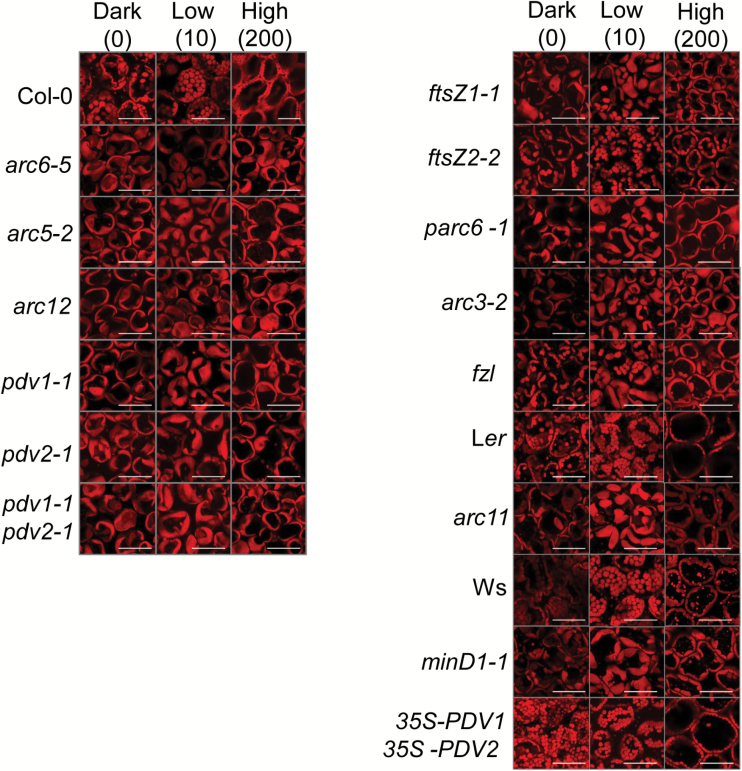
Confocal images showing chloroplast arrangement in mesophyll cells of the indicated genotypes exposed to different light levels. Dark-adapted plants were kept in 60 min of darkness or exposed to 60 min of low or high white light illumination. Leaf samples were then harvested for imaging. The red signal shows Chl autofluorescence and reveals the shapes of the chloroplasts. The numbers at the top indicate the light intensity in μmol photons m^–2^ s^–1^. Scale bars=50 μm.

Recent findings showed that mutants with 1–2 large chloroplasts failed to attain complete face or profile positioning when exposed to low- or high-light illuminations, respectively (Dutta *et al.*, 2015). To explore further the dependence of the capacity for photorelocation on chloroplast size, we assayed mutant lines with a range of chloroplast morphologies (Table 1; Supplementary Fig. S1) by imaging Chl autofluorescence in fixed leaf tissue using confocal microscopy. Figure 1 shows chloroplast positioning observed in palisade cells of leaves after 1 h of dark adaptation or after 1 h of exposure to low-intensity (10 µmol m^–2^ s^–1^) or high-intensity (200 µmol m^–2^ s^–1^) white light. As reported earlier (Königer and Bollinger, 2012; Dutta *et al.*, 2015), chloroplasts in the WT parental lines, Col-0, L*er*, and Ws, accumulated along the periclinal walls in low light and along the anticlinal walls in high light. No specific distribution pattern was observed in dark-adapted leaves. Light-dependent photorelocation was less distinguishable in the large-chloroplast mutants (Fig. 1, left panels; Table 1). Low-light-treated leaves showed an uneven repositioning of chloroplasts, with chloroplasts occupying both face and profile positions within the same mesophyll cell. High-light-treated leaves showed a higher proportion of chloroplasts occupying the profile position, with some still exhibiting a face position. Overall the distribution patterns were similar among all the large-chloroplast mutants studied. In contrast, the distribution patterns were quite diverse among the intermediate-chloroplast mutants (Fig. 1, right panels except bottom; Table 1). The *ftsZ2-2* and *arc11* mutants showed chloroplast arrangements resembling those in their respective Col-0 and L*er* WTs in both low and high light. Chloroplast distribution patterns in the remaining intermediate chloroplast mutants were more similar to those of the large-chloroplast mutants. The chloroplast distribution in the small-chloroplast genotype, *35S-PDV1 35S-PDV2* (Fig. 1, right bottom panels; Table 1), was indistinguishable from that of the Col-0 parent under all light regimes.

**Fig. 2.**
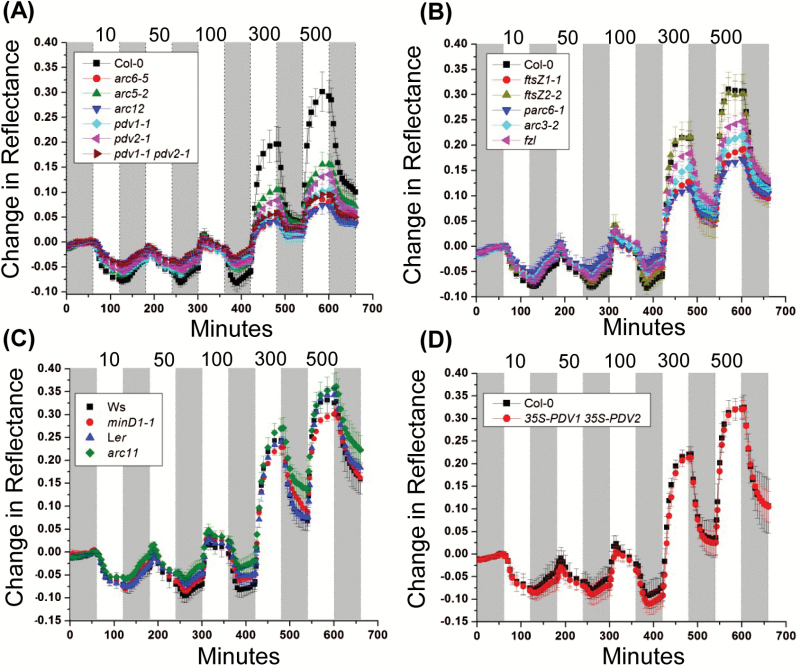
Chloroplast movement measurements in 18-day-old Arabidopsis plants of the indicated genotypes based on red light reflectance. Change in reflectance intensity versus time is shown during alternating 60 min periods of darkness (gray bars) or white light illumination (white bars) at the intensities indicated at the top of the graph (10–500 µmol m^–2^ s^–1^). For all data points, *n*=4–6 and error bars represent SDs. (A) Large-chloroplast mutants and the corresponding Col-0 wild type. (B) Intermediate-chloroplast mutants and the corresponding Col-0 wild type. (C) Intermediate-chloroplast mutants, *minD1-1* and *arc11* and their corresponding parental WTs, Ws and L*e*r, respectively. (D) *35S-PDV1 35S-PDV2* and the corresponding Col-0 wild type.

To better quantify the effect of chloroplast morphology on photorelocation, dark-adapted plants were assayed for accumulation and avoidance responses by measuring changes in the absorption of red light using reflectance imaging (Dutta *et al.*, 2015) in response to increasing intensities of white light (Fig. 2). The values and statistical analysis of the change in reflectance after 120 min at 10 µmol m^–2^ s^–1^ (maximum accumulation response) or 600 min at 500 µmol m^–2^ s^–1^ (maximum avoidance response) are shown in Table 2 and Supplementary Fig. S2.

**Table 2.** Change in reflectance values recorded at 120 min (accumulation response) and 600 min (avoidance response) in Arabidopsis plants of the indicated genotypes as described in Fig. 2

Genotype	Change in reflectance value ±SD (percentage of wild type)	
	120 min (accumulation response)	600 min (avoidance response)
Large chloroplasts		
Col-0	–0.074 ± 0.007 (100)	0.292 ± 0.031 (100)
*arc6-5*	–0.046 ± 0.006* (62.4)	0.079 ± 0.008* (27.2)
*arc5-2*	–0.063 ± 0.005* (84.6)	0.156 ± 0.021* (53.3)
*arc12*	–0.043 ± 0.007* (58.1)	0.072 ± 0.014* (24.8)
*pdv1-1*	–0.055 ± 0.007* (74.7)	0.103 ± 0.033* (35.3)
*pdv2-1*	–0.056 ± 0.008* (74.9)	0.153 ± 0.012* (46.3)
*pdv1-1 pdv2-1*	–0.042 ± 0.007* (56.3)	0.095 ± 0.004* (32.5)
Intermediate chloroplasts		
Col-0	–0.076 ± 0.005 (100)	0.307 ± 0.018 (100)
*ftsZ1-1*	–0.056 ± 0.004* (74.1)	0.190 ± 0.016* (62.1)
*ftsZ2-2*	–0.065 ± 0.009* (86.1)	0.301 ± 0.039 (98.1)
*parc6-1*	–0.049 ± 0.011* (65.6)	0.172 ± 0.009* (56.3)
*arc3-2*	–0.061 ± 0.004* (80.1)	0.218 ± 0.019* (71.1)
*fzl*	–0.066 ± 0.017* (87.0)	0.246 ± 0.017* (80.4)
Ws	–0.070 ± 0.014 (100)	0.325 ± 0.028 (100)
*minD1-1*	–0.075 ± 0.006 (105.9)	0.301 ± 0.012 (92.5)
L*er*	–0.071 ± 0.005 (100)	0.342 ± 0.014 (100)
*arc11*	–0.056 ± 0.005* (76.8)	0.358 ± 0.031 (104.5)
Small chloroplasts		
Col-0	–0.075 ± 0.018 (100)	0.323 ± 0.025 (100)
*35S-PDV1 35S-PDV2*	–0.081 ± 0.015 (107.5)	0.320 ± 0.019 (99.2)

For all data points, *n*=4–6 and error represents ±SD. Values marked with asterisks are significantly different from those in the relevant WT (Student’s *t*-test; *P*≤0.05).

All genotypes except *minD1-1* and *35S-PDV1 35S-PDV2* were attenuated in their photorelocation responses. Many of the division mutants with impaired movement had similar accumulation efficiencies (Fig. 2A, B; Table 2), with average reflectance changes ranging from ~74% to 85% of the change in the WT. *arc6-5*, *arc12*, *pdv1-1 pdv2-1*, and *parc6-1* were the exceptions, where the accumulation efficiency was ~56–65% that of the WT. In contrast, the maximum high-light avoidance efficiency differed more among the different groups of mutants. The large-chloroplast mutants showed severe impairment in avoidance responses, with most genotypes (*arc6-5*, *arc12*, *pdv1-1*, and *pdv1-1 pdv2-1*) showing reflectance changes approximating only 25–35% of the WT (Fig. 2A; Table 2; Supplementary Fig. S2). The other two mutants in this group (*arc5-2* and *pdv2-1*) showed ~50% attenuation in maximum avoidance responses. The results indicate that large-chloroplast genotypes impacted the high-light avoidance response more severely than the low-light accumulation response. Among the intermediate chloroplast mutants, *ftsZ1-1* and *parc6-1* had avoidance responses similar to those of most of the large-chloroplast mutants (55–60% of the WT). Interestingly, the *ftsZ2-2* and *arc11* mutants, which had impaired accumulation responses, showed avoidance efficiencies similar to those of their respective WTs, suggesting they may be more capable of avoiding excess photodamage under high-light conditions. A partial attenuation in avoidance response was observed in the remaining two members of this group (*arc3-2* and *fzl*), with reflectance changes averaging ~70–80% of that in the WT.

Based on the confocal and reflectance studies, we conclude that the accumulation and avoidance responses are differentially influenced by alterations in chloroplast size and shape, but that there is no simple relationship between chloroplast morphology and photorelocation phenotypes. For example, *arc6-5* and *arc12* are more impaired in their avoidance responses than *pdv1-1 pdv2-1* (Fig. 2A; Table 2; Supplementary Fig. S2) despite similarly enlarged chloroplast phenotypes (Supplementary Fig. S1B). Moreover, the fact that *arc11* in the L*er* background and *minD1-1* in the Ws background exhibit differences in their accumulation efficiencies, despite having lesions in the same gene and similar chloroplast morphologies (Zhang *et al.*, 2013) (Tables 1, 2; Fig. 2C; Supplementary Fig. S1), suggests that genetic background may influence photorelocation phenotypes (Kӧniger *et al.*, 2008).

### PSII efficiency and energy dissipation under high-light stress

Recently, we found that the impaired photorelocation capacity in three large-chloroplast mutants could not fully account for their high-light-induced photosynthetic phenotypes (Dutta *et al.*, 2015). To extend our analysis, we measured Chl fluorescence in all genotypes shown in Table 1 in a DEPI (Dutta *et al.*, 2015; Cruz *et al.*, 2016), which images fluorescence over all leaves in multiple intact plants. We used a 5 d light regime designed to simulate both static laboratory and dynamic field conditions and to reveal emergent phenotypes (Cruz *et al.*, 2016) (Supplementary Table S1). The regime included 2 d of constant light (Days 1 and 4, 100 μmol m^–2^ s^–1^), 1 d of ‘sinusoidal’ light (Day 2, with a maximum intensity of 500 μmol m^–2^ s^–1^), and 2 d of fluctuating light (Days 3 and 5, in which each light intensity step used on Day 2, termed here ‘ambient intensity’, was followed by a shorter exposure to twice that intensity, termed ‘fluctuating intensity’).

In these experiments, we analyzed the PSII quantum efficiency (Φ_II_), because this parameter is not influenced by differences in chloroplast photorelocation (Cazzaniga *et al.*, 2013; Dall’Osto *et al.*, 2014; Dutta *et al.*, 2015).

**Fig. 3.**
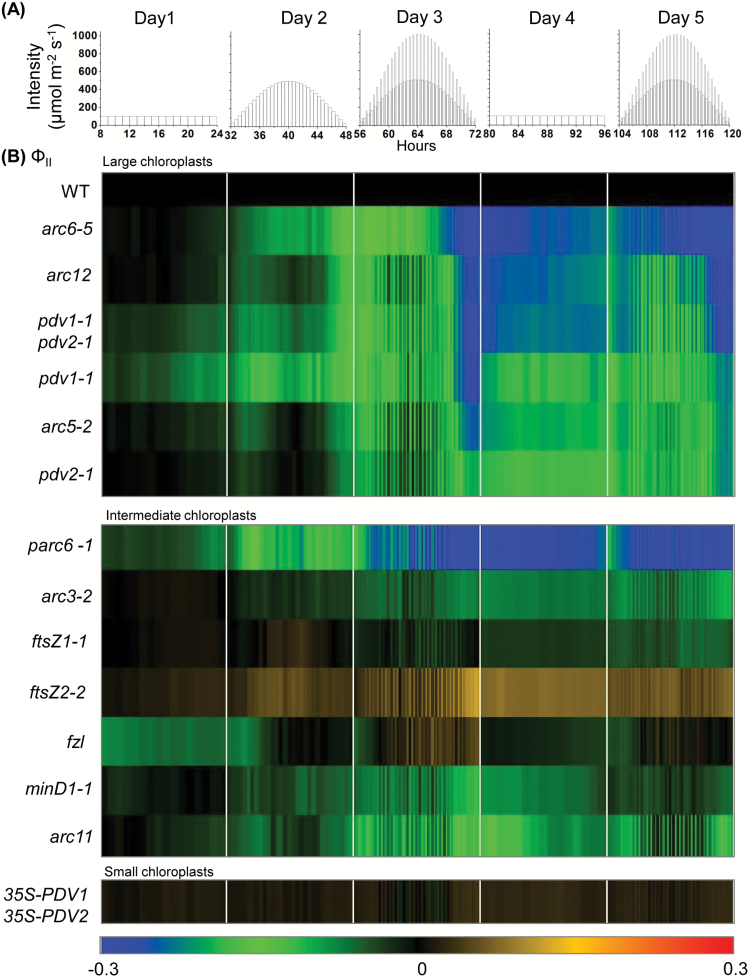
Changes in Φ_II_ in the indicated genotypes during a 5 d course of varying light. (A) Five day light regime. (B) Heat maps of Φ_II_. Measurements were taken every 60 min on Days 1 and 4, and at the end of each light interval on all other days. The color scale at the bottom depicts the log-fold increase (red) or decrease (blue) in values in the mutants normalized against values in corresponding parental lines (WT; black) at each time point. Each data point represents the mean of measurements from 6–10 plants. Raw data and statistical analysis are shown in Supplementary Figs S3–S7.

Figure 3 shows a heat map of the daily course of changes in Φ_II_ during the 5 d treatment in plants with different chloroplast phenotypes, expressed as log-fold changes relative to those in the relevant WT control. Raw, replicated data and statistical analyses for this study are presented in Supplementary Figs S3–S7. Overall, loss of Φ_II_ was correlated with the degree to which the chloroplasts deviated from normal size and number and was most pronounced under fluctuating light (Fig. 3). Plants with large chloroplasts (Table 1) showed the most severe Φ_II_ phenotypes. These can be categorized into (i) severe (*arc6-5*, *arc12*, and *pdv1-1 pdv2-1*), where the Φ_II_ phenotype accumulated to a significant level by the end of Day 3 and persisted on Days 4 and 5; (ii) moderately severe (*pdv1-1*, *arc5-2*), where the Φ_II_ phenotype recovered to some extent after Day 3 but increased gradually on Day 5; and (iii) less severe (*pdv2-1*), where a gradual increase in the Φ_II_ phenotype occurred over the 5 d light treatment.

Most mutants with intermediate-chloroplast phenotypes (*ftsZ1-1*, *ftsZ2-2*, *fzl*, *minD1-1*, and *arc11*) exhibited Φ_II_ phenotypes similar to those of the WT throughout the 5 d regime (Fig. 3; Supplementary Fig. S6). In *arc3-2*, Φ_II_ decreased on Day 5, implying that photoprotection was reduced in this mutant and might be further so if light treatments continued. In contrast to the other intermediate mutants, *parc6-1* showed a Φ_II_ phenotype resembling that of the most severe large-chloroplast mutants. No significant difference in Φ_II_ was recorded between the *35S-PDV1 35S-PDV2* line with small chloroplasts and the WT throughout the 5 d treatment (Fig. 3; Supplementary Fig. S6).

**Fig. 4.**
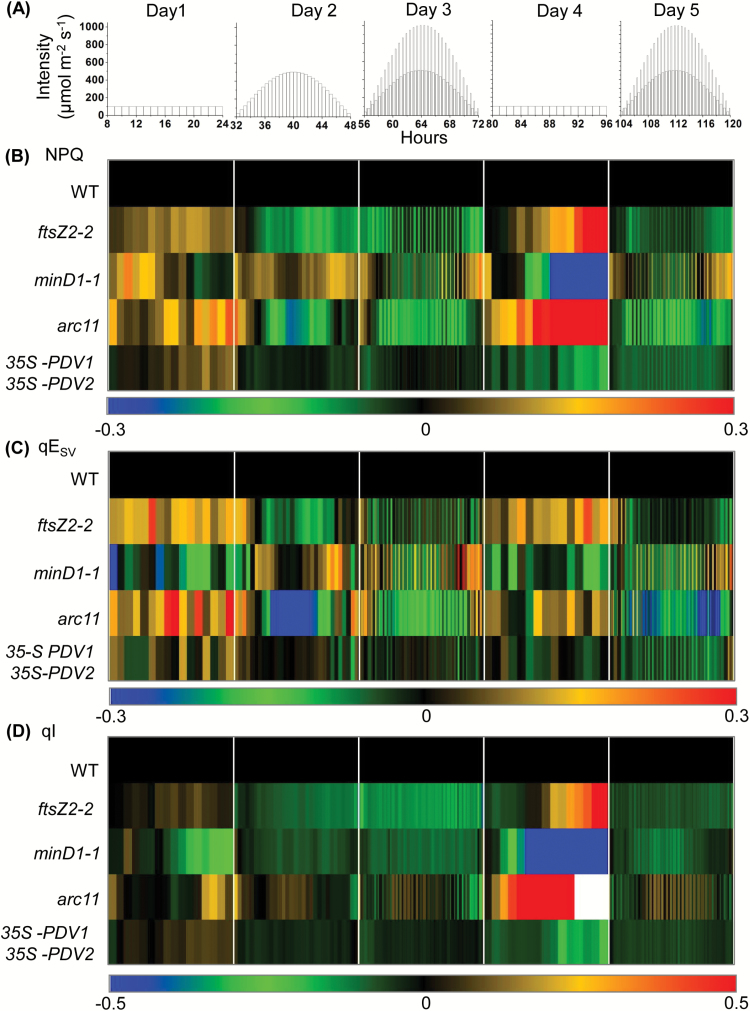
Changes in NPQ and its qE_SV_ and qI components in the indicated genotypes during a 5 d course of varying light. (A) Five day light regime; (B) NPQ; (C) qE_SV_; (D) qI. Measurements were taken every 60 min on Days 1 and 4, and at the end of each light interval on all other days. The color scale at the bottom depicts the log-fold increase (red) or decrease (blue) in values in the mutants normalized against values in the corresponding parental lines (WT; black) at each time point. The qI values in both L*er* and *arc11* were almost zero (white) towards the end of Day 4 (raw data in Supplementary Fig. S9). Each data point represents the mean of measurements from 6–10 plants. Raw data and statistical analysis are shown in Supplementary Figs S8–S11.

We also analyzed NPQ and its rapidly reversible, ΔpH- or energy-dependent component, qE (calculated as qE_SV_) (Krause and Jahns, 2003), and a longer lived photoinhibitory component, predominantly associated with photoinhibition, qI (Yamamoto and Kamite, 1972; Horton *et al.*, 1996; Li *et al.*, 2004; Murchie and Niyogi, 2011; Horton, 2012). Because chloroplast photorelocation efficiency influences NPQ measurements (Cazzaniga *et al.*, 2013; Dutta *et al.*, 2015), in these experiments the uncorrected fluorescence data (see below) could only be used to compare NPQ data for the set of genotypes in which photorelocation efficiencies (avoidance responses) were similar to that in the WT, namely *ftsZ2*-2, *minD1-1*, *arc11*, and *35S-PDV1 35S-PDV2* (Fig. 2; Table 2). Figure 4 displays heat map representations of NPQ, qE_SV_, and qI values for these genotypes. The corresponding raw data and statistical analyses are shown in Supplementary Figs S8–S11. Few significant differences in NPQ, qE_SV_, or qI were observed between *ftsZ2*-2, *minD1-1*, or *35S-PDV1 35S-PDV2* and the WT (Supplementary Figs S8–S11). The *arc11* mutant, which like *minD1-1* bears a mutation in *MinD1* and shares a similar chloroplast phenotype but in the L*er* background (Zhang *et al.*, 2013) (Supplementary Fig. S1B), showed somewhat lower NPQ and qE values compared with the WT on the first two high-light days (Days 2 and 3) (Fig. 4B, C; Supplementary Figs S9, S11B, C). On Day 4, *arc11* showed slightly higher NPQ values that could be attributed to a slight increase in qI, which nevertheless completely recovered by the end of the day (Fig. 4B, D; Supplementary Figs S9, S11B, D). These differences did not persist on Day 5, suggesting efficient acclimation of this mutant to high-light exposure.

Chl content was measured in rosette leaves prior to and following the 5 d light treatments. The WT exhibited the expected high-light responses, with a decreased Chl content and an increased Chl *a*/*b* ratio compared with untreated controls (Table 3), probably indicating preferential loss of antenna complexes following treatment (Bailey *et al.*, 2001; Phee *et al.*, 2004). These trends were also seen in the mutants, but with quantitative differences that fell into three categories. Prior to the 5 d experiment, the three large-chloroplast mutants (*arc6-5*, *arc5-2*, and *arc12*) already showed reduced Chl content (~82–87% that of Col-0), but this ratio remained about the same after the 5 d treatment. In contrast, one of the large-chloroplast mutants (*pdv1-1 pdv2-1*) and three intermediate-chloroplast mutants (*ftsZ1-1*, *arc3-2*, and *parc6-1*) had Chl contents close to that of the WT prior to the light treatment (99, 96, 94, and 92% of the WT for *pdv1-1 pdv2-1*, *ftsZ1-1*, *arc3-2*, and *parc6-1*, respectively), but displayed larger treatment-induced decreases (to 92, 88, 83, and 83% of the WT, respectively). Only two mutants (*arc6-5* and *parc6-1*) exhibited significantly elevated Chl *a*/*b* ratios (~40% higher than in the WT) after the 5 d treatment. These mutants and a few other intermediate-chloroplast mutants also showed small reductions (≤10%) in their Chl/carotenoid ratios (Table 3).

**Table 3.** Total Chl concentration, Chl *a*/*b* ratio, and Chl/carotenoid ratio (Chl/Car) in leaves of the indicated genotypes Pigment concentrations were measured before or immediately after the 5 d light treatment.

Genotype	Chl content (µg g^–1^ FW)		Chl *a*/*b*		Chl/Car	
Large chloroplasts	Before treatment	After 5 d light regime	Before treatment	After 5 d light regime	Before treatment	After 5 d light regime
Col-0	1190.9 ± 113.6	954.5 ± 27.3	2.9 ± 0.2	3.7 ± 0.2	4.2 ± 0.1	3.7 ± 0.1
*arc6-5*	1002.4 ± 62.94* (84.2)	781 ± 35.4* (81.8)	3.07 ± 0.16*	5.2 ± 0.6*	4.23 ± 0.01	3.4 ± 0.1*
*arc5-2*	979.7 ± 11.9* (82.3)	898.7 ± 15.6* (94.1)	3.2 ± 0.3	3.8 ± 0.1	4.2 ± 0.1	3.7 ± 0.04
*arc12*	1032.3 ± 14.9* (86.7)	841.8 ± 26.3* (88.2)	3.03 ± 0.2	4.2 ± 0.4*	4.2 ± 0.1	3.6 ± 0.1
*pdv1-1*	1095.7 ± 42.4 (92)	1002.9 ± 46.2 (105.1)	2.8 ± 0.1	4.5 ± 0.7	4.2 ± 0.1	3.7 ± 0.02
*pdv2-1*	1038.9 ± 62.6* (87.2)	869.1 ± 74 (91)	3.1 ± 0.3	3.9 ± 0.4	4.3 ± 0.1	3.7 ± 0.1
*pdv1-1pdv2-1*	1185.1 ± 83.8 (99.5)	878.6 ± 35.8* (92)	3.6 ± 0.4*	4.0 ± 0.5	4.2 ± 0.2	3.7 ± 0.1
Intermediate chloroplasts						
Col-0	1107.2 ± 41.7	893.7 ± 21.1	2.6 ± 0.1	3.4 ± 0.1	4.2 ± 0.1	3.9 ± 0.1
*ftsZ1-1*	1069.6 ± 43.2 (96.6)	793.6 ± 46.2* (88.8)	3.4 ± 0.2*	3.8 ± 0.2*	4.1 ± 0.03*	3.7 ± 0.1*
*ftsZ2-2*	1024.5 ± 76.9 (92.5)	893.1 ± 9.12 (99.9)	3.3 ± 0.3*	3.8 ± 0.1*	4.1 ± 0.05*	3.8 ± 0.03
*arc3-2*	1049.03 ± 23.3* (94.7)	744.8 ± 38.33* (83.3)	2.9 ± 0.3	4.0 ± 0.2*	4.3 ± 0.1	3.6 ± 0.03*
*fzl*	984.1 ± 62.9* (88.9)	788.5 ± 36.16* (88.2)	3.2 ± 0.3*	4.3 ± 0.1*	3.9 ± 0.1	3.5 ± 0.1*
*parc6 -1*	1024.9 ± 47.3* (92.6)	747.3 ± 41.54* (83.6)	3.1 ± 0.1*	4.7 ± 0.2*	4.3 ± 0.1	3.5 ± 0.04*
L*er*	1069.5 ± 55.7	945.9 ± 29.2	2.5 ± 0.2	4.1 ± 0.4	4.4 ± 0.05	3.7 ± 0.1
*arc11*	1009.2 ± 80.4 (94.4)	925.9 ± 16.3 (97.9)	2.9 ± 0.1*	4.4 ± 0.5	4.5 ± 0.1	3.6 ± 0.003*
Ws-2	1013 ± 86.2	795.9 ± 47.9	2.5 ± 0.2	4.02 ± 0.4	4.4 ± 0.2	3.7 ± 0.1
*minD1-1*	1092.4 ± 69.5 (107.8)	752.9 ± 71.04 (94.6)	2.8 ± 0.3	4.3 ± 0.1	4.4 ± 0.1	3.6 ± 0.1
Small Chloroplasts						
Col-0	1070.5 ± 64.2	847.3 ± 39.5	2.8 ± 0.04	3.3 ± 0.2	4.1 ± 0.02	3.6 ± 0.1
*35S-PDV1 35S-PDV2*	929.8 ± 60.3* (86.5)	832.5 ± 29.7 (98.2)	2.8 ± 0.1	3.3 ± 0.2	4.2 ± 0.1*	3.5 ± 0.1

For all data points, *n*=4–6 and error represents ±SD. Values marked with asterisks are significantly different from those in the relevant WT (Student’s *t*-test; *P*≤0.05).

### Analysis of chloroplast movement and photosynthesis in chloroplast division mutants with impaired photorelocation

Previous studies have shown that NPQ measurements are influenced by chloroplast movement (Cazzaniga *et al.*, 2013; Dutta *et al.*, 2015). Therefore, to disentangle the effects of chloroplast movement on NPQ, we employed dual imaging of red light reflectance and Chl fluorescence for simultaneous measurement of chloroplast movement and photosynthetic efficiency (Dutta *et al.*, 2015). This allowed us to estimate NPQ and its qE and qI components in genotypes with reduced chloroplast mobility, specifically all the large-chloroplast mutants and the intermediate mutants *ftsZ1-1*, *parc6-1*, *arc3-2*, and *fzl* (Fig. 2; Table 2). Based on the pronounced effect on Φ_II_ observed on Days 3 and 5 of the 5 d regime (Fig. 3), we chose the 16 h fluctuating light conditions for the single-day dual imaging experiments.

**Fig. 5.**
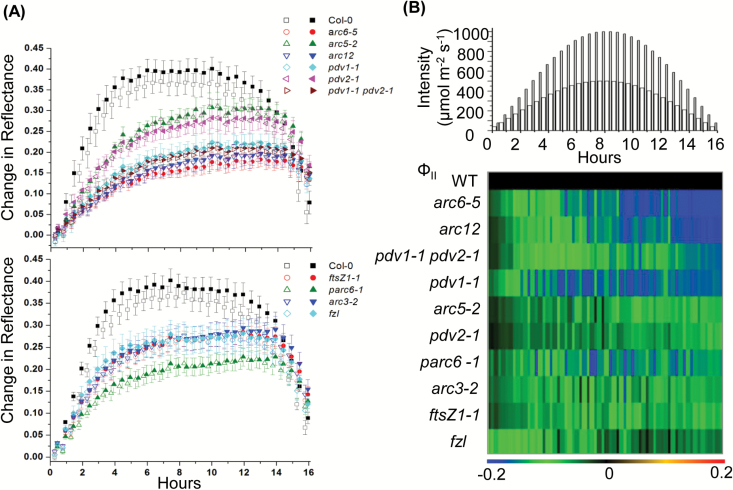
Chloroplast movement and Φ_II_ responses under fluctuating light. Three-week-old Arabidopsis plants of the indicated genotypes were subjected to the same white light regime shown for Day 3 in Fig. 4. (A) Chloroplast movement measured by reflectance in large- (upper panel) and intermediate- (lower panel) chloroplast mutants. Reflectance of pulsed red light was imaged from whole plants at each light intensity. The open and filled symbols correspond to alternating periods of ambient and fluctuating light, respectively. (B) Heat map comparing Φ_II_ responses in Col-0 (WT) and chloroplast division mutants. The light regime is illustrated at the top. Thick and thin bars represent ambient and fluctuating light intensities, respectively. The color scale at the bottom of each heat map depicts the log-fold increase (red) or decrease (blue) in values in the mutants normalized against values in the WT (black) at each time point. For all data points, *n*=4–6. The error bars in (A) represent the SD. Raw data and statistical analysis for the heat map (B) are shown in Supplementary Figs S12 and S14.

The time-courses of leaf reflectance changes are shown in Fig. 5A. The Col-0 WT showed a robust increase in reflectance after the start of the illumination period, saturating at ~6–8 h (maximum value ~0.4) and rapidly declining during the final few hours of illumination. In all the mutants studied, the onset kinetics of reflectance were slow and final recovery at the end of the illumination was significantly less than in the WT (Fig. 5A; Supplementary Table S2). The avoidance responses among the mutants followed patterns similar to those shown in Fig. 2, with *arc6-5*, *arc12*, *pdv1-1*, *pdv2-1*, *pdv1-1 pdv2-1*, and *parc6-1* showing maximum impairment in their high-light avoidance capacity (Supplementary Table S2).

Figure 5B shows a heat map representation of Φ_II_ values for all the mutant lines compared with values in the WT (Col-0). The corresponding raw, replicated data and statistical analyses are presented in Supplementary Figs S12A and S14. As expected from previous work (Baker, 2008), Φ_II_ decreased with increasing light intensity and recovered as the intensity decreased towards the end of the photoperiod (Supplementary Fig. S12A). In contrast to the results obtained on Day 3 of the 5 d experiment (Fig. 3B), in the single-day experiment the onset of the Φ_II_ phenotype occurred earlier and was generally more pronounced in most of the mutants, with *arc6-5* showing the most severe decrease in Φ_II_ (Fig. 5B; Supplementary Figs S12A, S14). This may be because plants had not received any prior high-light exposure in the single-day experiment. However, in *pdv1-1 pdv2-1*, Φ_II_ showed less susceptibility to fluctuating light in the single-day experiment, where the decrease in Φ_II_ was much less pronounced at the end of the day than on Day 3 of the 5 d experiment (Fig. 3B). *parc6-1* was also less susceptible to the single-day treatment and exhibited a slow recovery in Φ_II_ after mid-day. *pdv1-1* also showed a slight recovery. There was no such recovery in Φ_II_ in either *parc6-1* or *pdv1-1* on Day 3 of the 5 d experiment (Fig. 3B).

Overall, the results indicate that there is substantial variation among chloroplast division mutants in the susceptibility of Φ_II_ to short-term (Fig. 5B) or prolonged (Fig. 3) high-light stress, with large-chloroplast mutants and *parc6-1* generally exhibiting more pronounced Φ_II_ phenotypes.

**Fig. 6.**
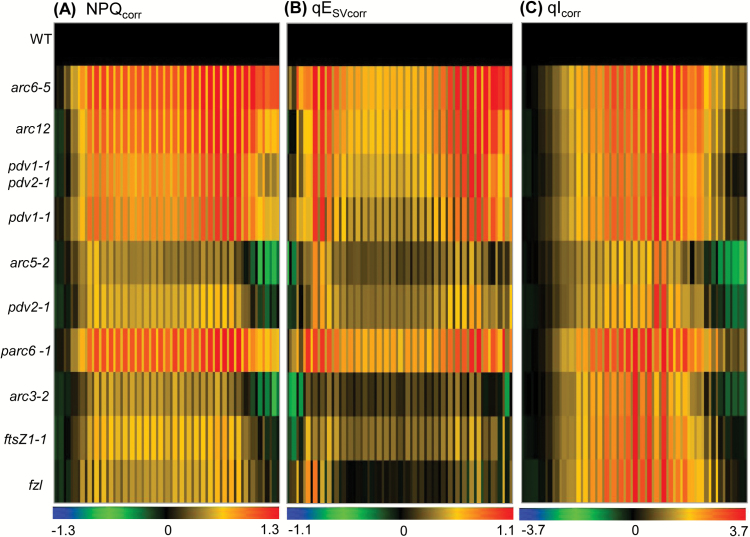
Heat map comparing non-photochemical quenching responses in Col-0 (WT) and chloroplast division mutants during a single day of fluctuating light as described for Fig. 5. Thick and thin bars represent ambient and fluctuating light intensities, respectively. The color scale at the bottom of each heat map depicts the log-fold increase (red) or decrease (blue) in values in the mutants normalized against values in the WT (black) at each time point. (A) NPQ, (B) qE_SV_, and (C) qI values corrected for chloroplast movement. Each data point represents the mean of 4–6 plants. Raw data and statistical analysis are shown in Supplementary Figs S12, S13, and S15.

To compare NPQ and its components qE and qI in plants with different chloroplast movement deficiencies, we corrected the calculation of NPQ for interference from chloroplast movement using the method described in Dutta *et al.* (2015). Figure 6 shows heat maps of the corrected values for non-photochemical quenching (NPQ_corr_), energy-dependent quenching (qE_SVcorr_), and long-lived NPQ that predominantly reflects photoinhibition (qI_corr_), expressed as log-fold changes compared with Col-0. Raw results (for both apparent and corrected values) and statistical analyses are presented in Supplementary Figs S12B–D, S13, and S15. All mutants had increased NPQ_corr_ compared with the WT during both ambient and fluctuating light (Fig. 6; Supplementary Fig. S15). The contribution of qI_corr_ to the overall NPQ_corr_ was higher than that of qE_SVcorr_ at mid-day when light intensities were higher, suggesting that photoinhibition was more pronounced at those intensities. Except for *arc6-5*, *arc12*, and *parc6-1*, all other mutants recovered by the end of the day (Fig. 6A; Supplementary Fig. S15A). *arc6-5* and *arc12* showed the highest NPQ_corr_ among all the large-chloroplast mutants. *parc6-1* was the only mutant in the intermediate-chloroplast group that showed significantly higher NPQ_corr_ than the WT, similar to *arc6-5* and *arc12*. All other intermediate mutants (*arc3-2*, *ftsZ1-1*, and *fzl*) showed moderately increased NPQ_corr_ compared with the WT, particularly at mid-day, which was primarily due to increased qI_corr_ (Fig. 6B, C; Supplementary Figs S13, S15). *arc5-2* and *pdv2-1* had the lowest NPQ_corr_ among the mutants studied. Our data indicate that although NPQ was affected in all the genotypes with impaired photorelocation, the distribution of NPQ and its components differed among mutants.

## Discussion

In this study, we investigated the influence of chloroplast morphology on high-light adaptation in Arabidopsis. Previous studies have shown that severe chloroplast division mutants display impaired chloroplast movement responses, leading to the hypothesis that their high-light-induced photosynthetic defects were due predominantly to their diminished chloroplast movement capacity (Jeong *et al.*, 2002; Königer *et al.*, 2008). However, we recently developed a non-invasive, whole-plant imaging approach to measure chloroplast photorelocation and Chl fluorescence parameters simultaneously (Dutta *et al.*, 2015). Our initial studies on chloroplast division mutants with only 1–2 drastically enlarged chloroplasts indicated that chloroplast size not only affected photorelocation but had additional effects on photosynthesis independent of the chloroplast movement defects (Dutta *et al.*, 2015). Here, we have extended our analysis to plants with a wider array of chloroplast morphology phenotypes (Table 1).

Consistent with a previous study (Königer *et al.*, 2008), we found a trend (Fig. 2) in which mutants with large or intermediate chloroplast sizes were defective in both the high-light avoidance response and low-light accumulation responses, but that the avoidance response was more strongly impacted. However, there were a few exceptions to these trends. Despite having somewhat larger chloroplasts than the Col-0 WT, the *ftsZ2-2* mutant, whose chloroplasts are fairly uniform in size and shape (McAndrew *et al.* 2008; Schmitz *et al.*, 2009) (Supplementary Fig. 1SB), was only impaired in its accumulation response (Table 2). Similarly, *parc6-1*, despite having intermediate chloroplast sizes on average (though with some large chloroplasts; Glynn *et al.*, 2009) exhibited stronger defects in the accumulation response than some large-chloroplast mutants (Table 2). In addition, *minD1-1*, which is in the Ws background, was unaffected in its accumulation response whereas *arc11*, which is in the L*er* background and has a chloroplast phenotype indistinguishable from that of *minD1-1* (Zhang *et al.*, 2013) (Supplementary Fig. 1SB), displayed an impaired accumulation response (Table 2). The latter results suggest that changes in chloroplast morphology may have distinct effects in different Arabidopsis accessions, which have been shown to exhibit differences in photorelocation responses (Königer *et al.*, 2008).

The large-chloroplast mutants showed fairly comparable defects in their avoidance responses, while the responses varied more among intermediate mutants with chloroplasts of more variable morphology (Table 2). One possible explanation could be related to the spatial distribution of components associated with the avoidance response. The avoidance response is thought to be mediated primarily by a pool of the blue light photoreceptor phot2 that is localized to the chloroplast outer envelope membrane (OEM) (Kong *et al.*, 2013*b*). Perception of strong blue light by OEM-localized phot2 results in the reorganization of short chloroplast-associated actin filaments (cp-actin); cp-actin disappears from the side of the chloroplast closest to the light, then accumulates on the distal side (Kong *et al.*, 2013*a*). The chloroplasts move in the direction of greater cp-actin accumulation (i.e. away from high light; Kadota *et al.*, 2009). The heterogeneity in chloroplast morphology in the intermediate division mutants may result in an altered distribution of phot2 in the OEM and/or more disorganized redistribution of cp-actin, thereby resulting in a more variable avoidance response. Study of cp-actin reorganization and localization of other chloroplast movement proteins in division mutants with chloroplasts of different sizes and shapes could provide mechanistic insight into the influence of chloroplast morphology on photorelocation efficiency.

Our previous studies have shown that the susceptibility of Φ_II_ to high-light stress in severe chloroplast division mutants was due more to increased chloroplast size itself than to the accompanying chloroplast movement defects (Dutta *et al.*, 2015). Using Arabidopsis plants with widely diverse chloroplast morphologies, we have now found that Φ_II_ is variably affected by changes in chloroplast size and shape, but is generally correlated with the severity of the morphology phenotype. For example, in the 5 d light regime experiment (Fig. 3), the mutants with 1–2 drastically enlarged chloroplasts (*arc6-5*, *arc12*, and *pdv1-1 pdv2-1*) showed a pronounced loss of Φ_II_ by the end of Day 3 as well as on Days 4 and 5, indicating long-term effects, whereas *pdv1-1* and *pdv2-1*, which have slightly less severe chloroplast phenotypes (Miyagishima *et al.*, 2006), showed overall milder Φ_II_ phenotypes. However, on Days 1 and 3, Φ_II_ was more affected in *pdv1-1* than in *pdv2-1* even though their chloroplast morphology phenotypes are very similar (Table 1; Supplementary Fig. S1B).

The nature of the photosynthetic phenotypes also differed between mutants. For example, in the 5 d treatment, *arc11* showed statistically significant decreases in qE compared with the WT on the high-light days (Days 2, 3, and 5), whereas qE was mostly unaffected on the same days in *ftsZ2-2* and *minD1-1* (Fig. 4C; Supplementary Figs S8, S9, S11C). Interestingly, the decreased qE in *arc11* was not accompanied by a significantly reduced PSII efficiency (Fig. 3B; Supplementary Figs S4, S6). This suggests the possibility of decreased capacity for the formation of a proton motive force through increased ATP synthase activity or reduced cyclic electron flow in this mutant (Kanazawa and Kramer, 2002; Munekage *et al.*, 2004), or alterations in other qE components, such as a decrease in xanthophyll cycle activity or reduced abundance of PsbS (Müller *et al.*, 2001, Li *et al.*, 2004, Kiss *et al.*, 2008). Alternatively, these differences may reflect qE responses specific to L*er*, the *arc11* parent, as natural variation in NPQ and other photosynthetic responses has been observed (Jung and Niyogi, 2009; Yin *et al.*, 2012).

While most of the mutants with intermediate-chloroplast phenotypes had Φ_II_ responses comparable with those in the WT throughout the 5 d treatment, *parc6-1* was a notable exception, showing a greater loss of Φ_II_ on Days 3–5 (Fig. 3; Supplementary Fig. S6). This may be partly because *parc6-1* has overall fewer and larger chloroplasts than other mutants categorized as intermediate, consistent with the greater severity of its chloroplast movement defects (Fig. 2; Table 2). However, all the large-chloroplast mutants except *arc6-5* had less pronounced Φ_II_ phenotypes than *parc6-1* (Fig. 3), suggesting that additional factors impacted overall photosynthetic efficiency in *parc6-1* and *arc6-5*. As ARC6 and PARC6 are paralogous proteins (Glynn *et al.*, 2009), it is possible that some related aspect of their functions contributed to the similarity of their mutant phenotypes despite their distinct functions in chloroplast division (Vitha *et al.*, 2003; Glynn *et al.*, 2008, 2009; Zhang *et al.*, 2016). Both *parc6-1* and *arc6-5* had drastically increased Chl *a*/*b* ratios (~40% higher than that of the WT) after the 5 d treatment (Table 3). This may suggest that changes in the components of the reaction center–antenna complex and antenna size might have contributed to the overall reduction of photosynthetic efficiency in these mutants (Tyystjärvi *et al.*, 1991; Falbel *et al.*, 1996).

Overall, the Φ_II_ data from the 5 d experiment suggest that plants with moderately oversized chloroplasts in their mesophyll cells are more capable of adjusting to high-light stress than plants with drastically enlarged chloroplasts. Further, the generally elevated susceptibility of Φ_II_ observed in the single-day fluctuating light regime compared with that on Day 3 of the 5 d experiment (Figs 3, 5B) suggests that plants with increased chloroplast size may be more affected by short-term high-light fluctuations that frequently occur under natural conditions.

As in the 5 d experiment, the kinetics and distribution of the components of NPQ in the single-day experiment were distinct in the mutants (Fig. 6; Supplementary Figs S13, S15). Photoinhibition (qI_corr_) was the main contributor to elevated NPQ_corr_ at mid-day, whereas qE_SVcorr_ was dominant in the early and later phases of the photoperiod. This result suggests that, although pH-regulated energy dissipation in the antenna of PSII (qE) is affected at the beginning and end of the periods of fluctuating light, photodamage of PSII outstripped repair at the saturating light intensities experienced at mid-day by these mutants. Accumulation of zeaxanthin associated with qE is also suggested to induce an additional, longer lived form of photoprotective quenching, termed qZ (Nilkens *et al.*, 2010). Therefore, the zeaxanthin level and hence elevated qZ may also have contributed to the NPQ_corr_ at mid-day in the division mutants. In contrast to the minimal Φ_II_ phenotype observed at mid-day in the intermediate-chloroplast mutants *arc3-2*, *ftsZ1-1*, and *fzl* (Fig. 5B; Supplementary Figs S12, S14), qI_corr_ was significantly higher in these mutants than in the WT at mid-day (Fig. 6C; Supplementary Figs S13C, S15C), indicating that their photosynthetic apparatuses are more prone to photodamage under saturating high-light intensities. As increased qI in high light corresponded with the degree to which chloroplast movements were suppressed in our previous study (Dutta *et al.*, 2015), the higher qI_corr_ observed in these mutants may be due to impairment in their avoidance responses (Figs 5A, 6C; Table 2). The Φ_II_ and NPQ responses of *arc5-2* and *pdv2-1* (Figs. 5B, 6; Supplementary Figs S14, S15) demonstrate that the losses in photosynthetic capacity in these mutants are predominantly due to impaired PSII operating efficiencies rather than being a consequence of photoinhibition under high light. The fact that we found few or no significant differences from the WT during the 5 d light regime in photosynthetic capacity in three of the mutants with intermediate-chloroplast phenotypes (*ftsZ2-2*, *arc11*, and *minD1-1*) (Figs 3, 4; Supplementary Figs S6, S8, S9, S11) suggests that other physiological functions of chloroplasts apart from photosynthesis may also be important for establishing the size, shape, and number of chloroplasts (Ohlrogge and Browse, 1995; Neuhaus and Emes, 2000; Lopez-Juez and Pyke, 2005; Bobik and Burch-Smith, 2015). The absence of any significant differences in chloroplast movement and photosynthetic efficiencies between *35S-PDV1 35S-PDV2* and Col-0 in our study raises further the fundamental question of why chloroplast division does not produce greater numbers of ‘small’ chloroplasts in mesophyll cells of Arabidopsis. It is also possible that the treatments used in this study were not sufficient to affect photosynthesis adversely in the *35S-PDV1 35S-PDV2* line.

Overall, the observed diversity of effects seen in the mutants implies that additional factors, beyond chloroplast size itself, contribute to decreased photosynthesis. It is not possible with the current data set to identify these factors unambiguously, but several possibilities are suggested by past work. In some of the mutants, asymmetric division results in heterogeneity in chloroplast shapes and sizes (Marrison *et al.*, 1999; Colletti *et al.*, 2000) (Table 1; Supplementary Fig. S1). In this case, the strength of the photosynthetic phenotype could be influenced by multiple factors including: (i) the size, shape, and distribution of the largest chloroplasts; (ii) competition between less efficient large chloroplasts and normal chloroplasts; and (iii) possible asymmetric distribution of internal components (e.g. thylakoids, Rubisco) among chloroplasts. In turn, these differences could directly or indirectly affect photosynthetic capacity, control, and regulation. Earlier work showed that several chloroplast division mutants have altered thylakoid organization and in some cases low mesophyll conductance compared with the WT (Pyke *et al.*, 1994; Austin and Webber, 2005; Weise *et al.*, 2015). Variations in the degree of thylakoid stacking, which have been observed in some chloroplast division mutants (Austin and Webber, 2005), could influence the relative distribution of the major photosynthetic complexes and consequently redox poise of the donor side of the electron transfer chain and susceptibility to PSII photoinhibition. *ARC5* (also called *DRP5B*) is required for peroxisome as well as chloroplast division (Zhang and Hu, 2010); thus alterations in peroxisome function could contribute to the photosynthetic phenotypes in *arc5-2*. In addition, differences in chloroplast shape (i.e. degree of folding, constriction, curvature, etc.) could influence the chloroplast surface-to-volume ratio, possibly limiting the rates of intracellular exchange of metabolites and leading to metabolic imbalances that, in turn, could limit the export of fixed carbon or alter the relative demands for ATP and NAPDH from the light reactions. We expect that any combination of these factors should affect the function of the light reactions by directly interfering with normal energy capture or by inducing feedback regulatory processes.

To conclude, our results suggest a number of possible explanations for the fact that plants with populations of enlarged or heterogeneous chloroplasts are rare or undescribed in nature. First, plant productivity may be affected by a reduction in optimum light absorption under low-light conditions (twilight, shade, overcast sky). Secondly, the reduction in PSII efficiency, particularly under fluctuating high light, may account for a significant loss of fitness in plants with larger or variably sized chloroplasts in a sunny environment. Thirdly, an increase in photoinhibition caused by suppression of the avoidance response may result in reduction in fitness in these plants. Taken together, this study demonstrates that it is necessary to maintain ‘normal’ chloroplast size and number in mesophyll cells of Arabidopsis for maximum photosynthetic performance under changing light conditions. Further functional and biochemical characterization under different stress conditions will provide additional insight into the effect of chloroplast morphology on plant performance.

## Supplementary data

Supplementary data are available at *JXB* online.

Fig. S1. Phenotypes of 30-day-old Arabidopsis plants used for this study.

Fig. S2. Heat maps showing the statistical significance of differences in reflectance values for data shown in Fig. 2 and Table 2.

Fig. S3. Raw PSII quantum yield (Φ_II_) data for plants with large-chloroplast phenotypes used to generate the results shown in Fig. 3.

Fig. S4. Raw PSII quantum yield (Φ_II_) data for plants with intermediate-chloroplast phenotypes used to generate the results shown in Fig. 3.

Fig. S5. Raw PSII quantum yield (Φ_II_) data for plants with small-chloroplast phenotypes used to generate the results shown in Fig. 3.

Fig. S6. Heat maps showing the statistical significance of differences in Φ_II_ between the WT and the indicated genotypes at each measurement time point for the data shown in Fig. 3 and Supplementary Figs S3–S5.

Fig. S7. Heat maps comparing the statistical significance of differences between Φ_II_ values in *arc6-5* and other large-chloroplast mutants (upper panels), and between *parc6-1* and other intermediate-chloroplast mutants (lower panels) at each time point for the data shown in Fig. 3.

Fig. S8. Raw NPQ (left), qEsv (middle), and qI (right) data for *ftsZ2-2* and its corresponding Col-0 wild type used to generate the results shown in Fig. 4.

Fig. S9. Raw NPQ (left), qEsv (middle), and qI (right) data for *minD1-1*, *arc11*, and their corresponding parental lines used to generate the results shown in Fig. 4.

Fig. S10. Raw NPQ (left), qEsv (middle), and qI (right) data for *35S-PDV1 35S-PDV-2* and its corresponding Col-0 wild type used to generate the results shown in Fig. 4.

Fig. S11. Heat maps showing the statistical significance of differences in photosynthetic parameters at each time point for the data shown in Fig. 4.

Fig. S12. Raw data for Φ_II_ and ‘apparent’ (traditional) NPQ, qE_SV_, and qI values for the experiment shown in Figs 5B and 6A–C, but uncorrected for chloroplast movements.

Fig. S13. Raw NPQ_corr_, qE_SVcorr_, and qI_corr_ data for the results shown in Fig. 6A–C.

Fig. S14. Heat map showing the statistical significance of differences in Φ_II_ between the WT and the indicated genotypes at each measurement time point for the data shown in Fig. 5B and Supplementary Fig. S12A.

Fig. S15. Heat maps showing the statistical significance of differences in photosynthetic parameters at each time point for the data shown in Fig. 6.

Table S1. Light conditions for the 5 d experiment shown in Fig. 3.

Table S2. Change in reflectance values from Fig. 5A recorded at the point of the maximum avoidance response and at the end of illumination in plants with large- and intermediate-chloroplast phenotypes.

## Supplementary Material

Supplementary Table S1

Supplementary Table S2

Supplementary Figure S1-S15

## References

[CIT0001] Augustynowicz J Gabryś H 1999 Chloroplast movements in fern leaves: correlation of movement dynamics and environmental flexibility of the species. Plant, Cell and Environment 22, 1239–1248.

[CIT0002] Austin JA II Webber AN 2005 Photosynthesis in *Arabidopsis thaliana* mutants with reduced chloroplast number. Photosynthesis Research 85, 373–384. 16170638 10.1007/s11120-005-7708-x

[CIT0003] Bailey S Walters RG Jansson S Horton P 2001 Acclimation of *Arabidopsis thaliana* to the light environment: the existence of separate low light and high light responses. Planta 213, 794–801. 11678285 10.1007/s004250100556

[CIT0004] Baker NR Oxborough K 2004 Chlorophyll fluorescence as a probe of photosynthetic productivity. In: Papageorgiou G Govindjee, eds. Chlorophyll a fluorescence: a signature of photosynthesis. Dordrecht: Springer Netherlands, 65–82.

[CIT0005] Baker NR 2008 Chlorophyll fluorescence: a probe of photosynthesis in vivo. Annual Review of Plant Biology 59, 89–113. 10.1146/annurev.arplant.59.032607.092759 18444897

[CIT0006] Bobik K Burch-Smith TM 2015 Chloroplast signaling within, between and beyond cells. Frontiers in Plant Science 6, 781. 26500659 10.3389/fpls.2015.00781 PMC4593955

[CIT0007] Bulychev AA Komarova AV 2015 Photoinduction of cyclosis-mediated interactions between distant chloroplasts. Biochimica et Biophysica Acta 1847, 379–389. 25615586 10.1016/j.bbabio.2015.01.004

[CIT0008] Cazzaniga S Dall’ Osto L Kong SG Wada M Bassi R 2013 Interaction between avoidance of photon absorption, excess energy dissipation and zeaxanthin synthesis against photooxidative stress in Arabidopsis. The Plant Journal 76, 568–579. 24033721 10.1111/tpj.12314

[CIT0009] Colletti KS Tattersall EA Pyke KA Froelich JE Stokes KD Osteryoung KW 2000 A homologue of the bacterial cell division site-determining factor MinD mediates placement of the chloroplast division apparatus. Current Biology 10, 507–516. 10801439 10.1016/s0960-9822(00)00466-8

[CIT0010] Crumpton-Taylor M Grandison S Png KM Bushby AJ Smith AM 2012 Control of starch granule numbers in Arabidopsis chloroplasts. Plant Physiology 158, 905–916. 22135430 10.1104/pp.111.186957 PMC3271777

[CIT0011] Cruz JA Savage LJ Zegarac R Hall CC Satoh-Cruz M Davis GA Kovac WK Chen J Kramer DM 2016 Dynamic environmental photosynthetic imaging reveals emergent phenotypes. Cell Systems 2, 365–377. 27336966 10.1016/j.cels.2016.06.001

[CIT0012] Davis PA Hangarter RP 2012 Chloroplast movement provides photoprotection to plants by redistributing PSII damage within leaves. Photosynthesis Research 112, 153–161. 22695784 10.1007/s11120-012-9755-4

[CIT0013] Dall’Osto L Cazzaniga S Wada M Bassi R 2014 On the origin of a slowly reversible fluorescence decay component in the *Arabidopsis npq4* mutant. Philosophical Transactions of the Royal Society B: Biological Sciences 369, 20130221. 10.1098/rstb.2013.0221 PMC3949386 24591708

[CIT0014] Dutta S Cruz JA Jiao Y Chen J Kramer DM Osteryoung KW 2015 Non-invasive, whole-plant imaging of chloroplast movement and chlorophyll fluorescence reveals photosynthetic phenotypes independent of chloroplast photorelocation defects in chloroplast division mutants. The Plant Journal 84, 428–442. 26332826 10.1111/tpj.13009

[CIT0015] Falbel TG Meehl JB Staehelin LA 1996 Severity of mutant phenotype in a series of chlorophyll-deficient wheat mutants depends on light intensity and the severity of the block in chlorophyll synthesis. Plant Physiology 112, 821–832. 8883392 10.1104/pp.112.2.821 PMC158007

[CIT0016] Gao H Kadirjan-Kalbach D Froehlich JE Osteryoung KW 2003 ARC5, a cytosolic dynamin-like protein from plants, is part of the chloroplast division machinery. Proceedings of the National Academy of Sciences, USA 100, 4328–4333. 10.1073/pnas.0530206100 PMC153092 12642673

[CIT0017] Gao H Sage TL Osteryoung KW 2006 FZL, an FZO-like protein in plants, is a determinant of thylakoid and chloroplast morphology. Proceedings of the National Academy of Sciences, USA 103, 6759–6764. 10.1073/pnas.0507287103 PMC1458954 16617119

[CIT0018] Glynn JM Froehlich JE Osteryoung KW 2008 *Arabidopsis* ARC6 coordinates the division machineries of the inner and outer chloroplast membranes through interaction with PDV2 in the intermembrane space. The Plant Cell 20, 2460–2470. 18812496 10.1105/tpc.108.061440 PMC2570736

[CIT0019] Glynn JM Miyagishima SY Yoder DW Osteryoung KW Vitha S 2007 Chloroplast division. Traffic 8, 451–461. 17451550 10.1111/j.1600-0854.2007.00545.x

[CIT0020] Glynn JM Yang Y Vitha S Schmitz AJ Hemmes M Miyagishima SY Osteryoung KW 2009 PARC6, a novel chloroplast division factor, influences FtsZ assembly and is required for recruitment of PDV1 during chloroplast division in Arabidopsis. The Plant Journal 59, 700–711. 19453460 10.1111/j.1365-313X.2009.03905.x

[CIT0021] Haupt W 1973 Role of light in chloroplast movement. BioScience 23, 289–296.

[CIT0022] Holtsmark I Lee S Lunde KA Auestad K Maple-Grødem J Møller SG 2013 Plastid division control: the PDV proteins regulate DRP5B dynamin activity. Plant Molecular Biology 82, 255–266. 23595201 10.1007/s11103-013-0059-7

[CIT0023] Hong Z Bednarek SY Blumwald E 2003 A unified nomenclature for *Arabidopsis* dynamin-related large GTPases based on homology and possible functions. Plant Molecular Biology 53, 261–265. 14750516 10.1023/b:plan.0000007000.29697.81

[CIT0024] Horton P 2012 Optimization of light harvesting and photoprotection: molecular mechanisms and physiological consequences. Philosophical Transactions of the Royal Society B: Biological Sciences 367, 3455–3465. 10.1098/rstb.2012.0069 PMC3497074 23148272

[CIT0025] Horton P Ruban AV Walters RG 1996 Regulation of light harvesting in green plants. Annual Review of Plant Physiology and Plant Molecular Biology 47, 655–684. 10.1146/annurev.arplant.47.1.655 15012304

[CIT0026] Itoh R Fujiwara M Nagata N Yoshida S 2001 A chloroplast protein homologous to the eubacterial topological specificity factor minE plays a role in chloroplast division. Plant Physiology 127, 1644–1655. 11743109 PMC133569

[CIT0027] Jarvis P López-Juez E 2013 Biogenesis and homeostasis of chloroplasts and other plastids. Nature Reviews. Molecular Cell Biology 14, 787–802. 24263360 10.1038/nrm3702

[CIT0028] Jeong WJ Park YI Suh K Raven JA Yoo OJ Liu JR 2002 A large population of small chloroplasts in tobacco leaf cells allows more effective chloroplast movement than a few enlarged chloroplasts. Plant Physiology 129, 112–121. 12011343 10.1104/pp.000588 PMC155876

[CIT0029] Jung HS Niyogi KK 2009 Quantitative genetic analysis of thermal dissipation in Arabidopsis. Plant Physiology 150, 977–986. 19339502 10.1104/pp.109.137828 PMC2689978

[CIT0030] Kadota A Yamada N Suetsugu N Hirose M Saito C Shoda K Ichikawa S Kagawa T Nakano A Wada M 2009 Short actin-based mechanism for light-directed chloroplast movement in *Arabidopsis*. Proceedings of the National Academy of Sciences, USA 106, 13106–13111. 10.1073/pnas.0906250106 PMC2722281 19620714

[CIT0031] Kanazawa A Kramer DM 2002 *In vivo* modulation of nonphotochemical exciton quenching (NPQ) by regulation of the chloroplast ATP synthase. Proceedings of the National Academy of Sciences, USA 99, 12789–12794. 10.1073/pnas.182427499 PMC130538 12192092

[CIT0032] Kasahara M Kagawa T Oikawa K Suetsugu N Miyao M Wada M 2002 Chloroplast avoidance movement reduces photodamage in plants. Nature 420, 829–832. 12490952 10.1038/nature01213

[CIT0033] Kiss AZ Ruban AV Horton P 2008 The PsbS protein controls the organization of the photosystem II antenna in higher plant thylakoid membranes. Journal of Biological Chemistry 283, 3972–3978. 18055452 10.1074/jbc.M707410200

[CIT0034] Kong SG Arai Y Suetsugu N Yanagida T Wada M 2013 *a* Rapid severing and motility of chloroplast-actin filaments are required for the chloroplast avoidance response in *Arabidopsis*. The Plant Cell 25, 572–590. 23404888 10.1105/tpc.113.109694 PMC3608779

[CIT0035] Kong SG Suetsugu N Kikuchi S Nakai M Nagatani A Wada M 2013 *b* Both phototropin 1 and 2 localize on the chloroplast outer membrane with distinct localization activity. Plant and Cell Physiology 54, 80–92. 23161859 10.1093/pcp/pcs151

[CIT0036] Königer M Bollinger N 2012 Chloroplast movement behavior varies widely among species and does not correlate with high light stress tolerance. Planta 236, 411–426. 22395438 10.1007/s00425-012-1619-9

[CIT0037] Königer M Delamaide JA Marlow ED Harris GC 2008 *Arabidopsis thaliana* leaves with altered chloroplast numbers and chloroplast movement exhibit impaired adjustments to both low and high light. Journal of Experimental Botany 59, 2285–2297. 18468985 10.1093/jxb/ern099 PMC2423661

[CIT0038] Krause GH Jahns P 2003 Pulse amplitude modulated chlorophyll fluorometry and its application in plant science. In: Green B Parson WW, eds. Light-harvesting antennas in photosynthesis. Dordrecht: Springer Netherlands, 373–399.

[CIT0039] Li XP Gilmore AM Caffarri S Bassi R Golan T Kramer D Niyogi KK 2004 Regulation of photosynthetic light harvesting involves intrathylakoid lumen pH sensing by the PsbS protein. Journal of Biological Chemistry 279, 22866–22874. 15033974 10.1074/jbc.M402461200

[CIT0040] Lopez-Juez E Pyke KA 2005 Plastids unleashed: their development and their integration in plant development. International Journal of Developmental Biology 49, 557–577. 16096965 10.1387/ijdb.051997el

[CIT0041] Maple J Chua NH Møller SG 2002 The topological specificity factor AtMinE1 is essential for correct plastid division site placement in *Arabidopsis*. The Plant Journal 31, 269–277. 12164807 10.1046/j.1365-313x.2002.01358.x

[CIT0042] Maple J Møller SG 2007 Interdependency of formation and localisation of the Min complex controls symmetric plastid division. Journal of Cell Science 120, 3446–3456. 17855384 10.1242/jcs.010215

[CIT0043] Maple J Vojta L Soll J Møller SG 2007 ARC3 is a stromal Z-ring accessory protein essential for plastid division. EMBO Reports 8, 293–299. 17304239 10.1038/sj.embor.7400902 PMC1808034

[CIT0044] Marrison JL Rutherford SM Robertson EJ Lister C Dean C Leech RM 1999 The distinctive roles of five different *ARC* genes in the chloroplast division process in *Arabidopsis*. The Plant Journal 18, 651–662. 10417716 10.1046/j.1365-313x.1999.00500.x

[CIT0045] McAndrew RS Olson BJ Kadirjan-Kalbach DK Chi-Ham CL Vitha S Froehlich JE Osteryoung KW 2008 *In vivo* quantitative relationship between plastid division proteins FtsZ1 and FtsZ2 and identification of ARC6 and ARC3 in a native FtsZ complex. Biochemical Journal 412, 367–378. 18284374 10.1042/BJ20071354

[CIT0046] Miyagishima SY Froehlich JE Osteryoung KW 2006 PDV1 and PDV2 mediate recruitment of the dynamin-related protein ARC5 to the plastid division site. The Plant Cell 18, 2517–2530. 16998069 10.1105/tpc.106.045484 PMC1626610

[CIT0047] Müller P Li XP Niyogi KK 2001 Non-photochemical quenching. A response to excess light energy. Plant Physiology 125, 1558–1566. 11299337 10.1104/pp.125.4.1558 PMC1539381

[CIT0048] Munekage Y Hashimoto M Miyake C Tomizawa K Endo T Tasaka M Shikanai T 2004 Cyclic electron flow around photosystem I is essential for photosynthesis. Nature 429, 579–582. 15175756 10.1038/nature02598

[CIT0049] Murchie EH Niyogi KK 2011 Manipulation of photoprotection to improve plant photosynthesis. Plant Physiology 155, 86–92. 21084435 10.1104/pp.110.168831 PMC3075776

[CIT0050] Nakanishi H Suzuki K Kabeya Y Miyagishima SY 2009 Plant-specific protein MCD1 determines the site of chloroplast division in concert with bacteria-derived MinD. Current Biology 19, 151–156. 19135368 10.1016/j.cub.2008.12.018

[CIT0051] Neuhaus HE Emes MJ 2000 Nonphotosynthetic metabolism in plastids. Annual Review of Plant Physiology and Plant Molecular Biology 51, 111–140. 10.1146/annurev.arplant.51.1.111 15012188

[CIT0052] Nilkens M Kress E Lambrev P Miloslavina Y Müller M Holzwarth AR Jahns P 2010 Identification of a slowly inducible zeaxanthin-dependent component of non-photochemical quenching of chlorophyll fluorescence generated under steady-state conditions in *Arabidopsis*. Biochimica et Biophysica Acta 1797, 466–475. 20067757 10.1016/j.bbabio.2010.01.001

[CIT0053] Nott A Jung HS Koussevitzky S Chory J 2006 Plastid-to-nucleus retrograde signaling. Annual Review of Plant Biology 57, 739–759. 10.1146/annurev.arplant.57.032905.105310 16669780

[CIT0054] Ohlrogge J Browse J 1995 Lipid biosynthesis. The Plant Cell 7, 957–970. 7640528 10.1105/tpc.7.7.957 PMC160893

[CIT0055] Okazaki K Kabeya Y Suzuki K Mori T Ichikawa T Matsui M Nakanishi H Miyagishima SY 2009 The PLASTID DIVISION1 and 2 components of the chloroplast division machinery determine the rate of chloroplast division in land plant cell differentiation. The Plant Cell 21, 1769–1780. 19567705 10.1105/tpc.109.067785 PMC2714929

[CIT0056] Olson BJ Wang Q Osteryoung KW 2010 GTP-dependent heteropolymer formation and bundling of chloroplast FtsZ1 and FtsZ2. Journal of Biological Chemistry 285, 20634–20643. 20421292 10.1074/jbc.M110.122614 PMC2898327

[CIT0057] Osteryoung KW Pyke KA 2014 Division and dynamic morphology of plastids. Annual Review of Plant Biology 65, 443–472. 10.1146/annurev-arplant-050213-035748 24471836

[CIT0058] Osteryoung KW Stokes KD Rutherford SM Percival AL Lee WY 1998 Chloroplast division in higher plants requires members of two functionally divergent gene families with homology to bacterial ftsZ. The Plant Cell 10, 1991–2004. 9836740 10.1105/tpc.10.12.1991 PMC143974

[CIT0059] Phee BK Cho JH Park S Jung JH Lee YH Jeon JS Bhoo SH Hahn TR 2004 Proteomic analysis of the response of *Arabidopsis* chloroplast proteins to high light stress. Proteomics 4, 3560–3568. 15478214 10.1002/pmic.200400982

[CIT0060] Porra RJ 2002 The chequered history of the development and use of simultaneous equations for the accurate determination of chlorophylls a and b. Photosynthesis Research 73, 149–156. 16245116 10.1023/A:1020470224740

[CIT0061] Pyke KA 2013 Divide and shape: an endosymbiont in action. Planta 237, 381–387. 22910876 10.1007/s00425-012-1739-2

[CIT0062] Pyke KA Leech RM 1991 Rapid image analysis screening procedure for identifying chloroplast number mutants in mesophyll cells of *Arabidopsis thaliana* (L.) Heynh. Plant Physiology 96, 1193–1195. 16668319 10.1104/pp.96.4.1193 PMC1080914

[CIT0063] Pyke KA Leech RM 1992 Chloroplast division and expansion is radically altered by nuclear mutations in *Arabidopsis thaliana*. Plant Physiology 99, 1005–1008. 16668963 10.1104/pp.99.3.1005 PMC1080576

[CIT0064] Pyke KA Leech RM 1994 A genetic analysis of chloroplast division and expansion in *Arabidopsis thaliana*. Plant Physiology 104, 201–207. 12232072 10.1104/pp.104.1.201 PMC159178

[CIT0065] Pyke KA Rutherford SM Robertson EJ Leech RM 1994 *arc6*, a fertile *Arabidopsis* mutant with only two mesophyll cell chloroplasts. Plant Physiology 106, 1169–1177. 12232400 10.1104/pp.106.3.1169 PMC159646

[CIT0066] Raghavendra AS Padmasree K 2003 Beneficial interactions of mitochondrial metabolism with photosynthetic carbon assimilation. Trends in Plant Science 8, 546–553. 14607100 10.1016/j.tplants.2003.09.015

[CIT0067] Schmitz AJ, Glynn JM, Olson BJ, Stokes KD, Osteryoung KW 2009 *Arabidopsis* FtsZ2-1 and FtsZ2-2 are functionally redundant, but FtsZ-based plastid division is not essential for chloroplast partitioning or plant growth and development. Molecular Plant 2, 1211–1222. 10.1093/mp/ssp077 19995726

[CIT0068] Schneider CA, Rasband WS, Eliceiri KW 2012. NIH Image to ImageJ: 25 years of image analysis. Nature Methods 9, 671–675. 10.1038/nmeth.2089 PMC5554542 22930834

[CIT0069] Senn G 1908 Die Gestalts-und Lageveränderung der Pflanzen-Chromatophoren. Engelmann: Leipzig.

[CIT0070] Shimada H Koizumi M Kuroki K Mochizuki M Fujimoto H Ohta H Masuda T Takamiya K 2004 ARC3, a chloroplast division factor, is a chimera of prokaryotic FtsZ and part of eukaryotic phosphatidylinositol-4-phosphate 5-kinase. Plant and Cell Physiology 45, 960–967. 15356321 10.1093/pcp/pch130

[CIT0071] Smith AG Johnson CB Vitha S Holzenburg A 2010 Plant FtsZ1 and FtsZ2 expressed in a eukaryotic host: GTPase activity and self-assembly. FEBS Letters 584, 166–172. 19925792 10.1016/j.febslet.2009.11.044

[CIT0072] Sztatelman O Waloszek A Banaś AK Gabryś H 2010 Photoprotective function of chloroplast avoidance movement: *in vivo* chlorophyll fluorescence study. Journal of Plant Physiology 167, 709–716. 20172619 10.1016/j.jplph.2009.12.015

[CIT0073] TerBush AD Osteryoung KW 2012 Distinct functions of chloroplast FtsZ1 and FtsZ2 in Z-ring structure and remodeling. Journal of Cell Biology 199, 623–637. 23128242 10.1083/jcb.201205114 PMC3494859

[CIT0074] Timmis JN Ayliffe MA Huang CY Martin W 2004 Endosymbiotic gene transfer: organelle genomes forge eukaryotic chromosomes. Nature Reviews. Genetics 5, 123–135. 10.1038/nrg1271 14735123

[CIT0075] Trojan A Gabrys H 1996 Chloroplast distribution in *Arabidopsis thaliana* (L.) depends on light conditions during growth. Plant Physiology 111, 419–425. 12226297 10.1104/pp.111.2.419 PMC157851

[CIT0076] Tyystjärvi E Koivuniemi A Kettunen R Aro EM 1991 Small light-harvesting antenna does not protect from photoinhibition. Plant Physiology 97, 477–483. 16668423 10.1104/pp.97.2.477 PMC1081031

[CIT0077] Vitha S Froehlich JE Koksharova O Pyke KA van Erp H Osteryoung KW 2003 ARC6 is a J-domain plastid division protein and an evolutionary descendant of the cyanobacterial cell division protein Ftn2. The Plant Cell 15, 1918–1933. 12897262 10.1105/tpc.013292 PMC167179

[CIT0078] Vitha S McAndrew RS Osteryoung KW 2001 FtsZ ring formation at the chloroplast division site in plants. Journal of Cell Biology 153, 111–120. 11285278 10.1083/jcb.153.1.111 PMC2185535

[CIT0079] Wada M Grolig F Haupt W 1993 Light-oriented chloroplast positioning. Contribution to progress in photobiology. Journal of Photochemistry and Photobiology B: Biology 17, 3–25.

[CIT0080] Wada M 2013 Chloroplast movement. Plant Science 210, 177–182. 23849124 10.1016/j.plantsci.2013.05.016

[CIT0081] Weise SE Carr DJ Bourke AM Hanson DT Swarthout D Sharkey TD 2015 The arc mutants of *Arabidopsis* with fewer large chloroplasts have a lower mesophyll conductance. Photosynthesis Research 124, 117–126. 25733184 10.1007/s11120-015-0110-4

[CIT0082] Wellburn AR 1994 The spectral determination of chlorophylls a and b, as well as total carotenoids, using various solvents with spectrophotometers of different resolution. Journal of Plant Physiology 144, 307–313.

[CIT0083] Yamamoto HY Kamite L 1972 The effects of dithiothreitol on violaxanthin de-epoxidation and absorbance changes in the 500-nm region. Biochimica et Biophysica Acta 267, 538–543. 5047136 10.1016/0005-2728(72)90182-x

[CIT0084] Yin L Fristedt R Herdean A 2012 Photosystem II function and dynamics in three widely used *Arabidopsis thaliana* accessions. PLoS One 7, e46206. 23029436 10.1371/journal.pone.0046206 PMC3460815

[CIT0085] Yoder DW Kadirjan-Kalbach D Olson BJ Miyagishima SY Deblasio SL Hangarter RP Osteryoung KW 2007 Effects of mutations in Arabidopsis *FtsZ1* on plastid division, FtsZ ring formation and positioning, and FtsZ filament morphology in vivo. Plant and Cell Physiology 48, 775–791. 17468127 10.1093/pcp/pcm049

[CIT0086] Yoshida Y Mogi Y TerBush AD Osteryoung KW 2016 Chloroplast FtsZ assembles into a contractible ring via tubulin-like heteropolymerization. Nature Plants 2, 16095. 27322658 10.1038/nplants.2016.95

[CIT0087] Zhang M Chen C Froehlich JE TerBush AD Osteryoung KW 2016 Roles of Arabidopsis PARC6 in coordination of the chloroplast division complex and negative regulation of FtsZ assembly. Plant Physiology 170, 250–262. 26527658 10.1104/pp.15.01460 PMC4704591

[CIT0088] Zhang M Schmitz AJ Kadirjan-Kalbach DK Terbush AD Osteryoung KW 2013 Chloroplast division protein ARC3 regulates chloroplast FtsZ-ring assembly and positioning in *Arabidopsis* through interaction with FtsZ2. The Plant Cell 25, 1787–1802. 23715471 10.1105/tpc.113.111047 PMC3694706

[CIT0089] Zhang X Hu J 2010 The *Arabidopsis* chloroplast division protein DYNAMIN-RELATED PROTEIN5B also mediates peroxisome division. The Plant Cell 22, 431–442. 20179140 10.1105/tpc.109.071324 PMC2845408

