# Supplementary material for: Variations in chloroplast movement and chlorophyll fluorescence among chloroplast division mutants under light stress
Source: J Exp Bot. 2017 Jun 22;68(13):3541–55. doi: 10.1093/jxb/erx203 (PMC5853797; doi:10.1093/jxb/erx203)
Supplement: Supplementary Table S1 [file erx203_suppl_supplementary_table_s1.pdf]

Day 1	Day 2	Day 3	Day 4	Day 5
100	39	39	100	39
	80	78		78
	123	80		80
	167	161		161
	210	123		123
	253	246		246
	294	167		167
	333	333		333
	370	210		210
	402	420		420
	431	253		253
	455	506		506
	475	294		294
	489	588		588
	497	333		333
	500	667		667
	500	370		370
	497	739		739
	489	402		402
	475	805		805
	455	431		431
	431	862		862
	402	455		455
	370	911		911
	333	475		475
	294	949		949
	253	489		489
	210	977		977
	167	497		497
	123	994		994
	80	500		500
	39	1000		1000
		500		500
		1000		1000
		497		497
		994		994
		489		489
		977		977
		475		475
		949		949
		455		455
		911		911
		431		431
		862		862
		402		402
		805		805
		370		370
		739		739
		333		333
		667		667
		294		294
		588		588
		253		253
		506		506
		210		210
		420		420
		167		167
		333		333
		123		123
		246		246
		80		80
		161		161
		39		39
		78		78

Table S1. Light conditions for the five-day long experiment shown in Figure 3.
The numbers in the table show the white light intensities (μmol m^{-2} s^{-1}) during every interval of the 16-h light period for the experiment shown in Figure 3.
