# Supplementary material for: Variations in chloroplast movement and chlorophyll fluorescence among chloroplast division mutants under light stress
Source: J Exp Bot. 2017 Jun 22;68(13):3541–55. doi: 10.1093/jxb/erx203 (PMC5853797; doi:10.1093/jxb/erx203)
Supplement: Supplementary Table S2 [file erx203_suppl_supplementary_table_s2.pdf]

Genotype	Change in reflectance Value ±SD	
	Max avoidance response	End of illumination
Large chloroplasts		
Col-0	0.397 ± 0.027 (100)	0.078 ± 0.027
arc6-5	0.182 ± 0.016***** (45.84)	0.133 ± 0.014*****
arc12	0.196 ± 0.022***** (49.37)	0.146 ± 0.017*****
pdv1-1 pdv2-1	0.213 ± 0.012***** (53.65)	0.151 ± 0.013*****
pdv1-1	0.222 ± 0.021***** (55.91)	0.136 ± 0.015*****
arc5-2	0.305 ± 0.020***** (76.82)	0.165 ± 0.012*****
pdv2-1	0.283 ± 0.026***** (71.28)	0.169 ± 0.024*****
Intermediate chloroplasts		
Col-0	0.401 ± 0.026 (100)	0.088 ± 0.013
parc6-1	0.223 ± 0.021***** (55.61)	0.127 ± 0.017*****
arc3-2	0.293 ± 0.029* (73.07)	0.154 ± 0.022*
ftsZ1-1	0.284 ± 0.020***** (70.82)	0.142 ± 0.019*****
fzl	0.281 ± 0.034***** (70.07)	0.121 ± 0.017*****

Table S2. Change in reflectance values from Figure 5A recorded at the point of the maximum avoidance response and at the end of illumination in plants with large (upper panel) and intermediate (lower panel) chloroplast phenotypes as described in Figure 5A. For all data points, n = 4-6 and error represents ± SD. Values marked with asterisks are significantly different (Student's t-test; $P \leq 0.05$). The number in parentheses represents the percentage of reflectance recorded relative to that in the relevant WT background.
