# Supplementary material for: Variations in chloroplast movement and chlorophyll fluorescence among chloroplast division mutants under light stress
Source: J Exp Bot. 2017 Jun 22;68(13):3541–55. doi: 10.1093/jxb/erx203 (PMC5853797; doi:10.1093/jxb/erx203)
Supplement: Supplementary Figure S1-S15 [file erx203_suppl_supplementary_figure_s1_s15.pdf]

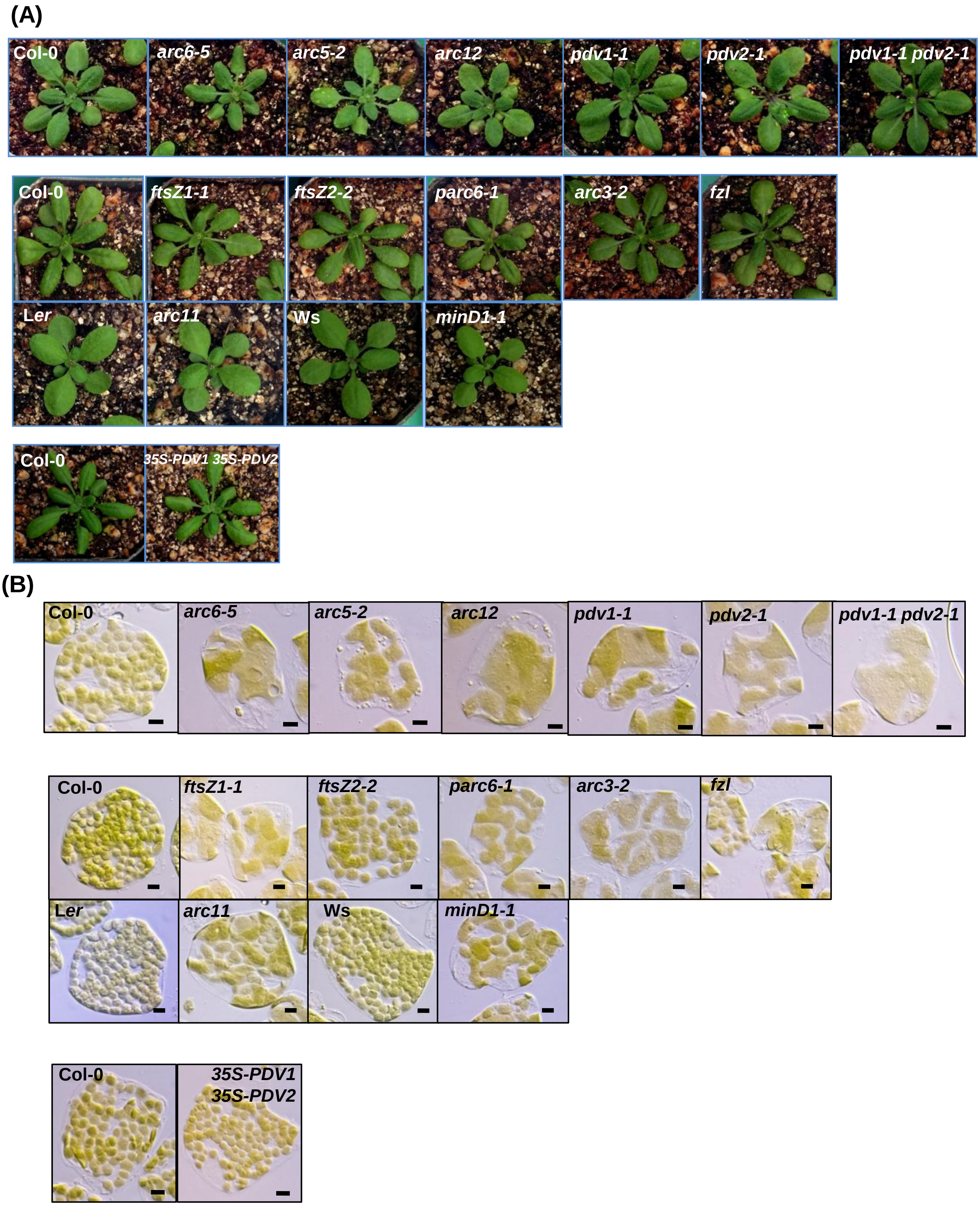

Fig. S1. Phenotypes of 30-day-old Arabidopsis plants used for this study. Plants were grown side-by-side in a 16/8-h light/dark. Upper, middle and lower panels depict plants with large, intermediate and small chloroplasts, respectively. Images from the wild-type parents (Col-0, L*er*, or Ws) are shown to the left of the relevant genotypes. (A) Whole-plant phenotypes. (B) Chloroplast morphology phenotypes.. Images are not true color; differences in the intensity of the green color are due to slightly different white balance and/or intensity. Scale bars, 10 µm.

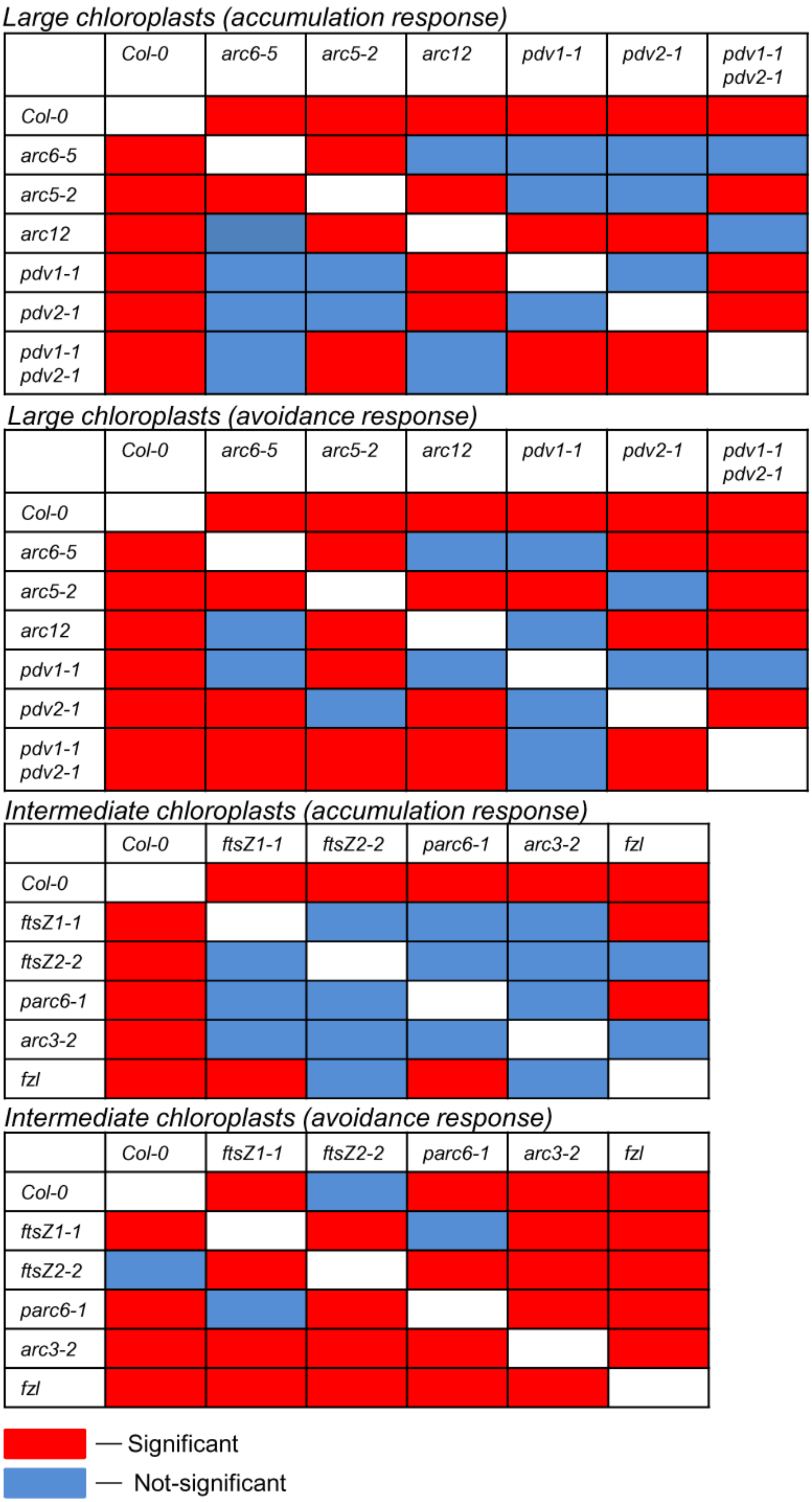

Fig. S2. Heat maps showing the statistical significance of differences in reflectance values for data shown in Figure 2 and Table 2. Reflectance values were recorded at 120 min (accumulation response) and 600 min (avoidance response) in plants with large or intermediate chloroplast morphologies. Mutants that showed significant difference in their accumulation and/or avoidance responses compared to those in the corresponding parental line are displayed in the heat maps. Differences were evaluated using the unpaired, two-tailed t-test. P values ≤ 0.05 were taken as statistically significant. Red and blue boxes represent statistically significant and non-significant differences, respectively.

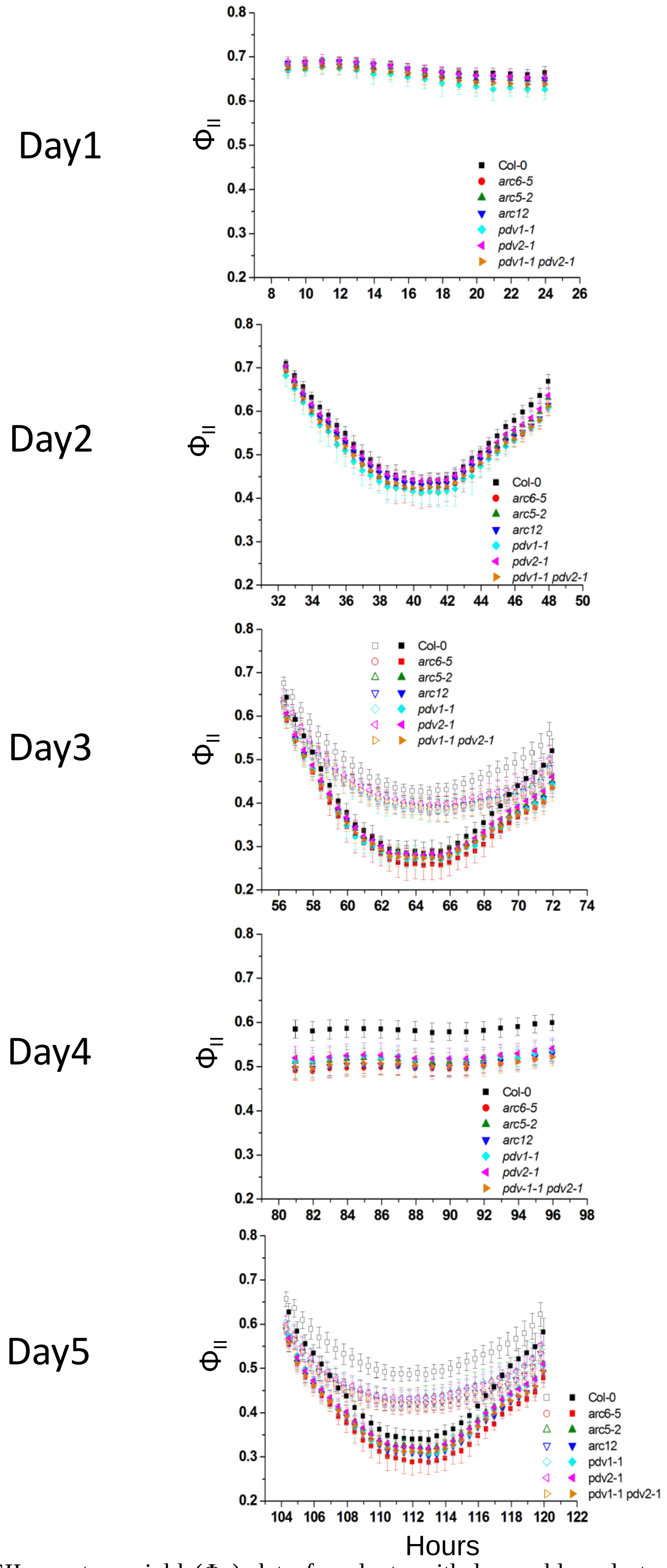

Fig. S3. Raw PSII quantum yield (Φ_{II}) data for plants with large-chloroplast phenotypes used to generate results shown in Figure 3. The open and closed symbols on Days 3 and 5 correspond to alternating periods of ambient and fluctuating light, respectively. Each data point represents the mean of 6-10 plants; error bars represent SD.

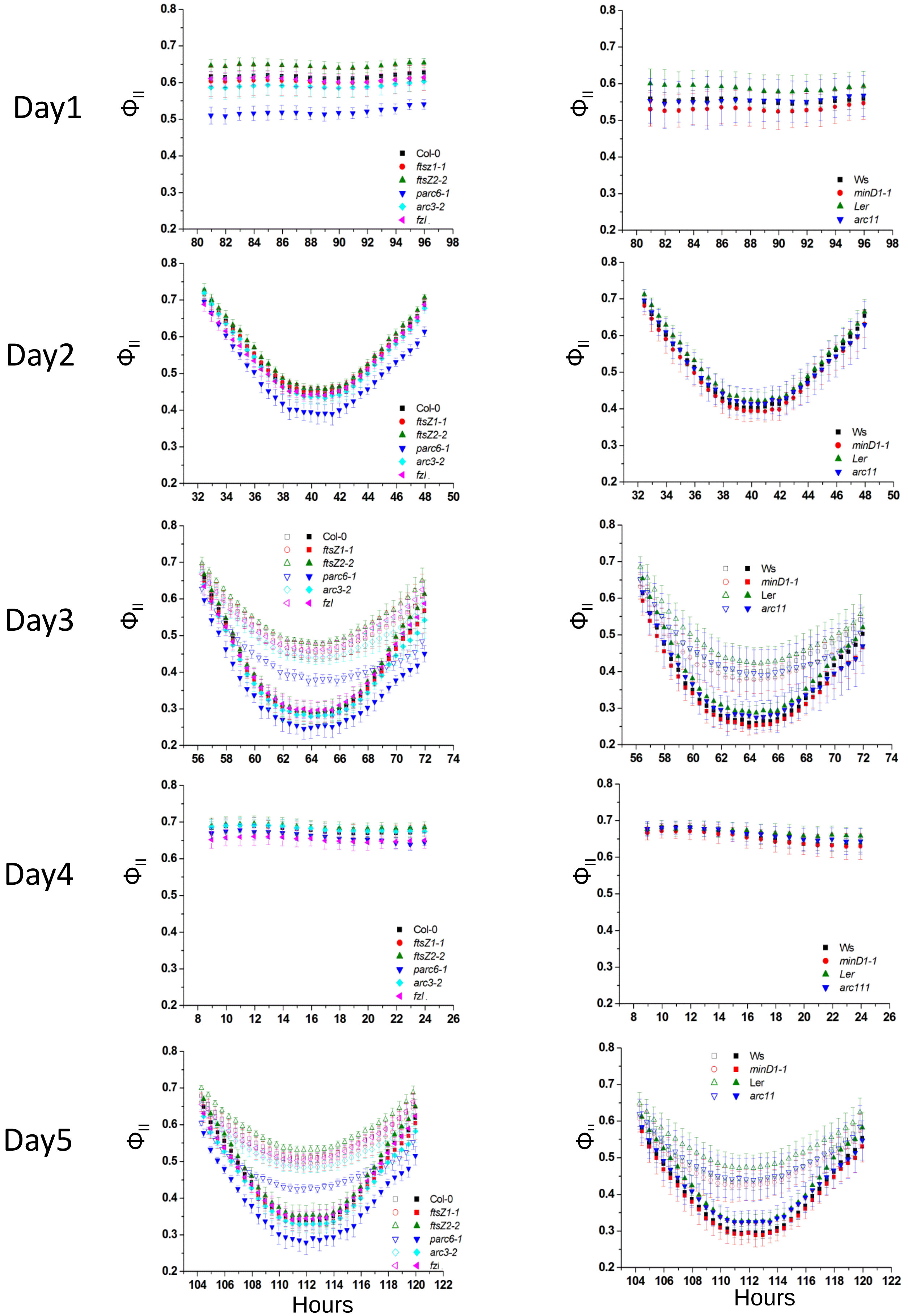

Fig. S4. Raw PSII quantum yield (Φ_{II}) data for plants with intermediate-chloroplast phenotypes used to generate results shown in Figure 3. The open and closed symbols on Days 3 and 5 correspond to alternating periods of ambient and fluctuating light, respectively. Each data point represents the mean of 6-10 plants; error bars represent SD.

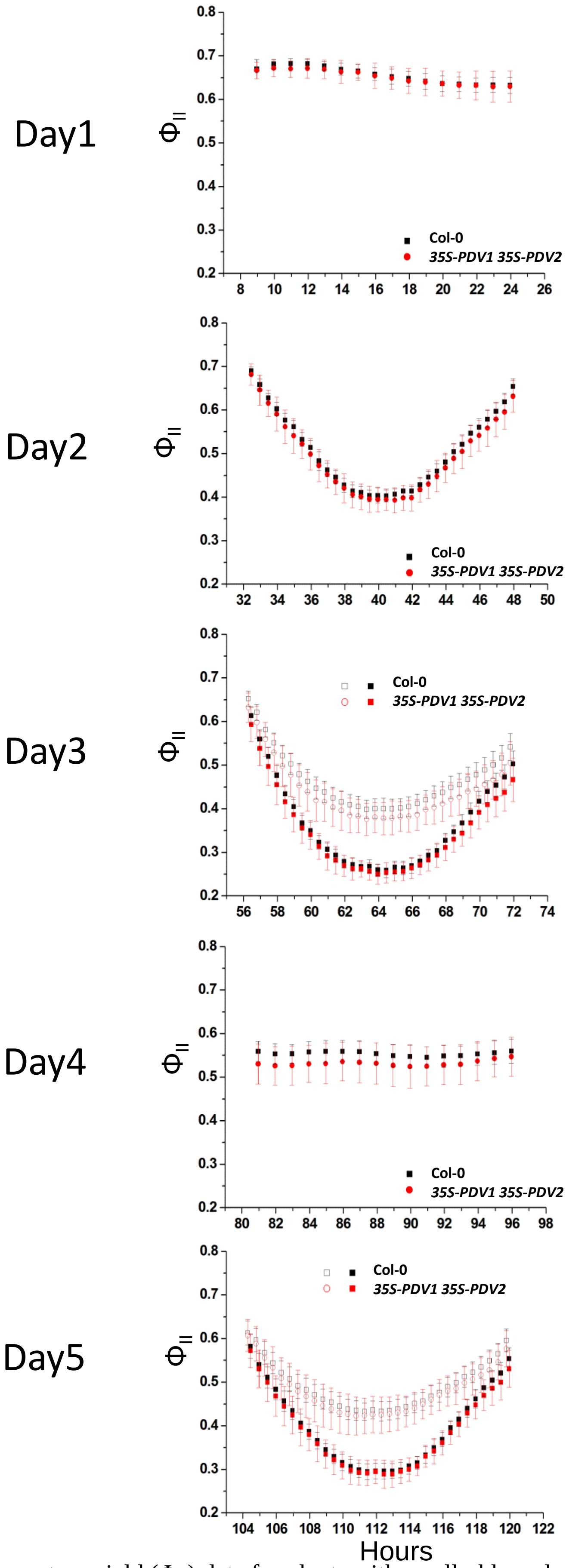

Fig. S5. Raw PSII quantum yield (Φ_{II}) data for plants with small-chloroplast phenotypes used to generate results shown in Figure 3. The open and closed symbols on Days 3 and 5 correspond to alternating periods of ambient and fluctuating light, respectively. Each data point represents the mean of 6-10 plants; error bars represent SD.

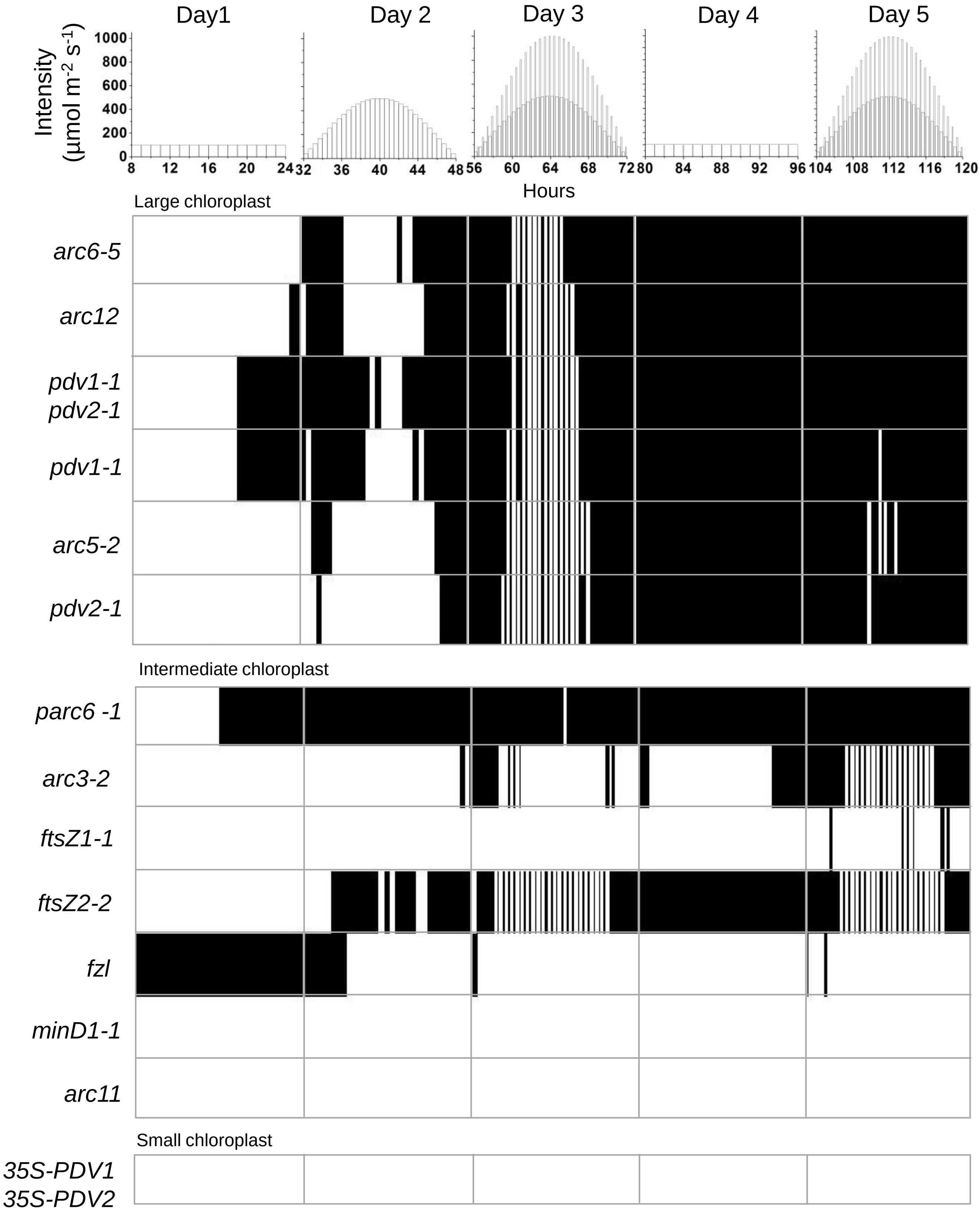

Fig. S6. Heat map showing the statistical significance of differences in Φ_{II} between WT and indicated genotypes at each measurement time point for the data shown in Figures 3 and S3-5. Significance was evaluated using the unpaired, two-tailed t-test. P values ≤ 0.05 were taken as statistically significant (black stripes). The light regime is shown at top.

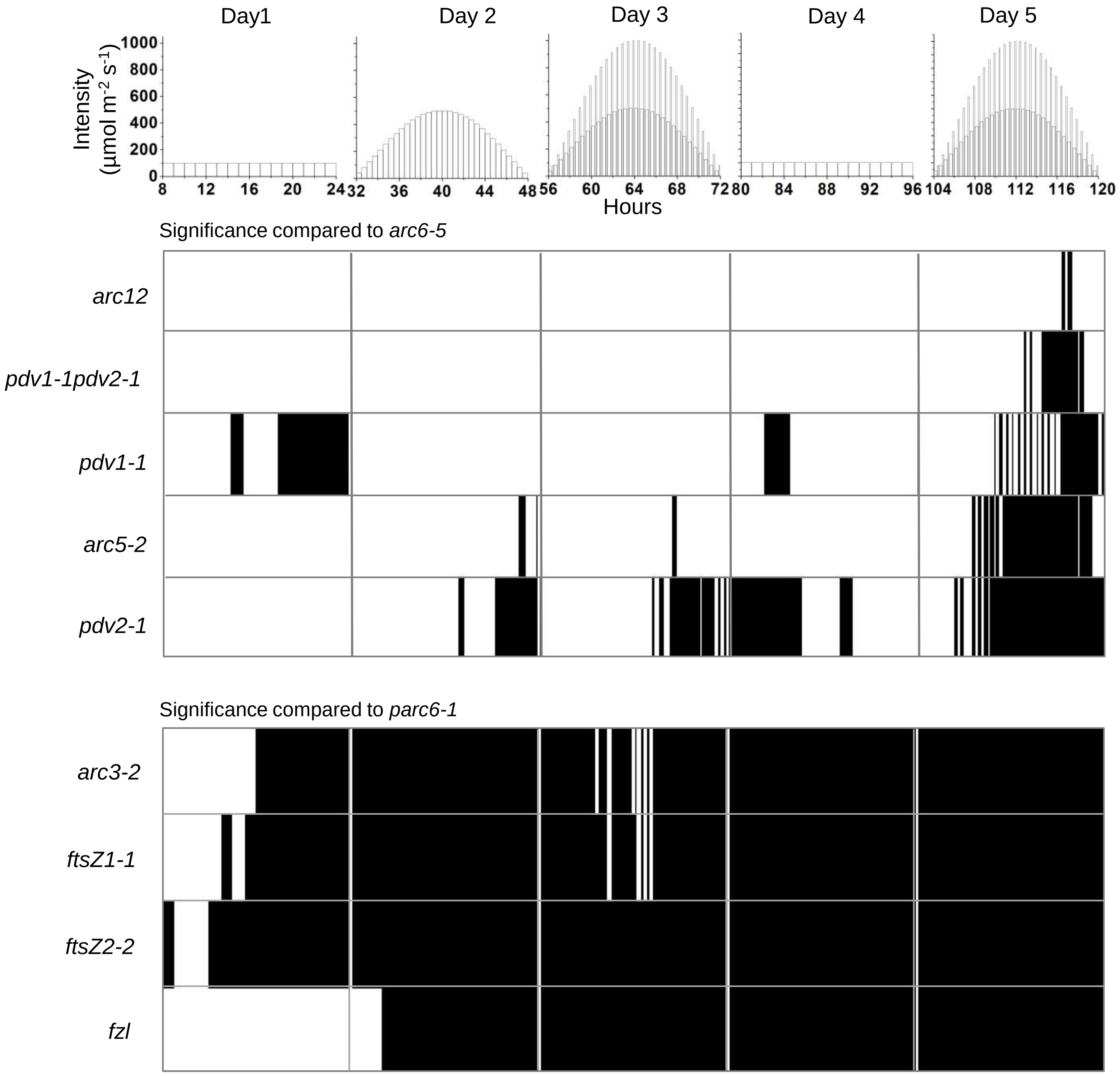

Fig. S7. Heat maps comparing the statistical significance of differences between Φ_{II} values in *arc6-5* and other large-chloroplast mutants (upper panels), and between *parc6-1* and other intermediate-chloroplast mutants (lower panels) at each time point for data shown in Figure 3. Differences were evaluated using the unpaired, two-tailed t-test. P values ≤ 0.05 were taken as statistically significant (black stripes). The light regime is shown at top.

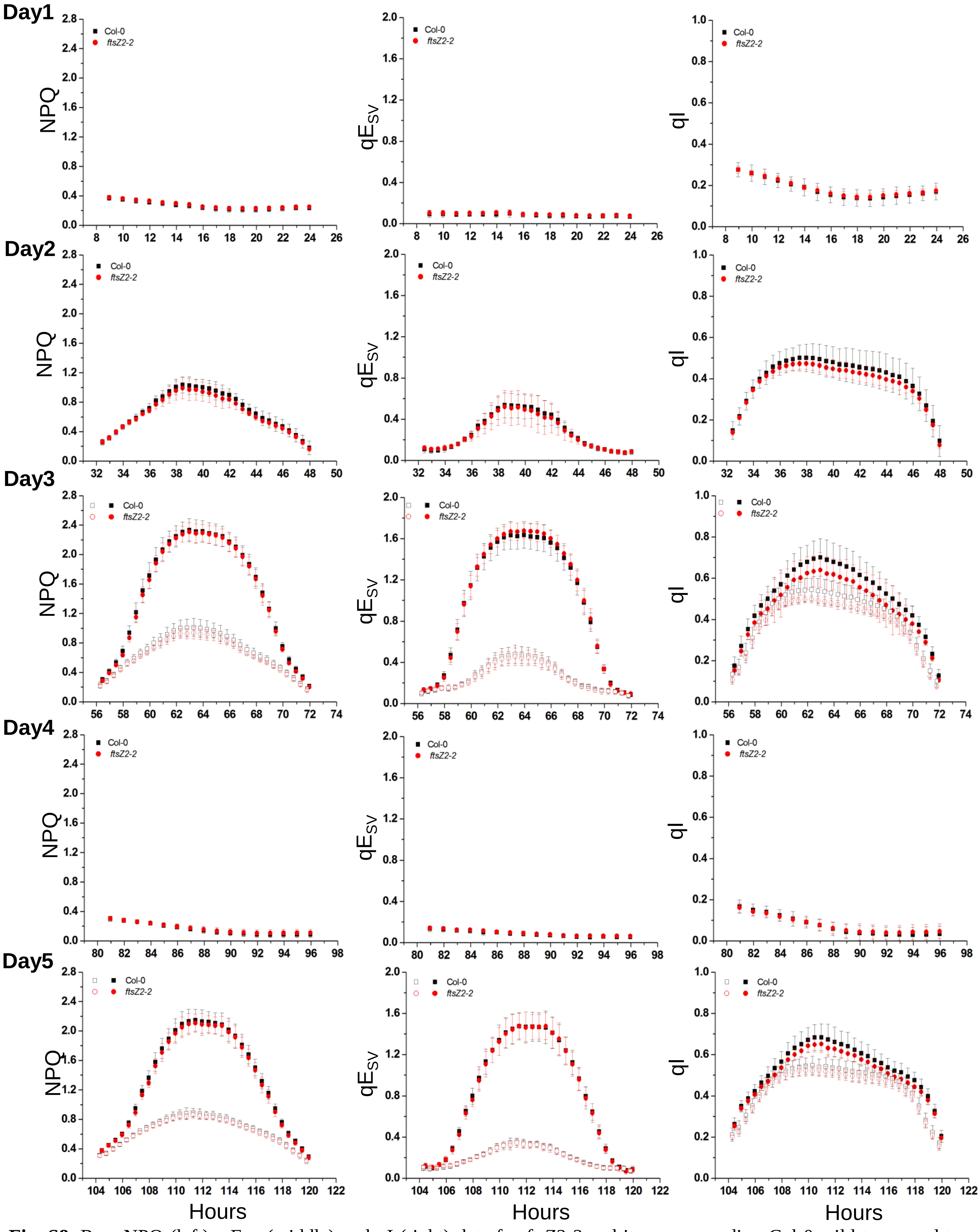

Fig. S8. Raw NPQ (left), qEsv (middle) and qI (right) data for *ftsZ2-2* and its corresponding Col-0 wild type used to generate results shown in Figure 4. The open and closed symbols on Days 3 and 5 correspond to alternating periods of ambient and fluctuating light, respectively. Each data point represents the mean of 6-10 plants; error bars represent SD.

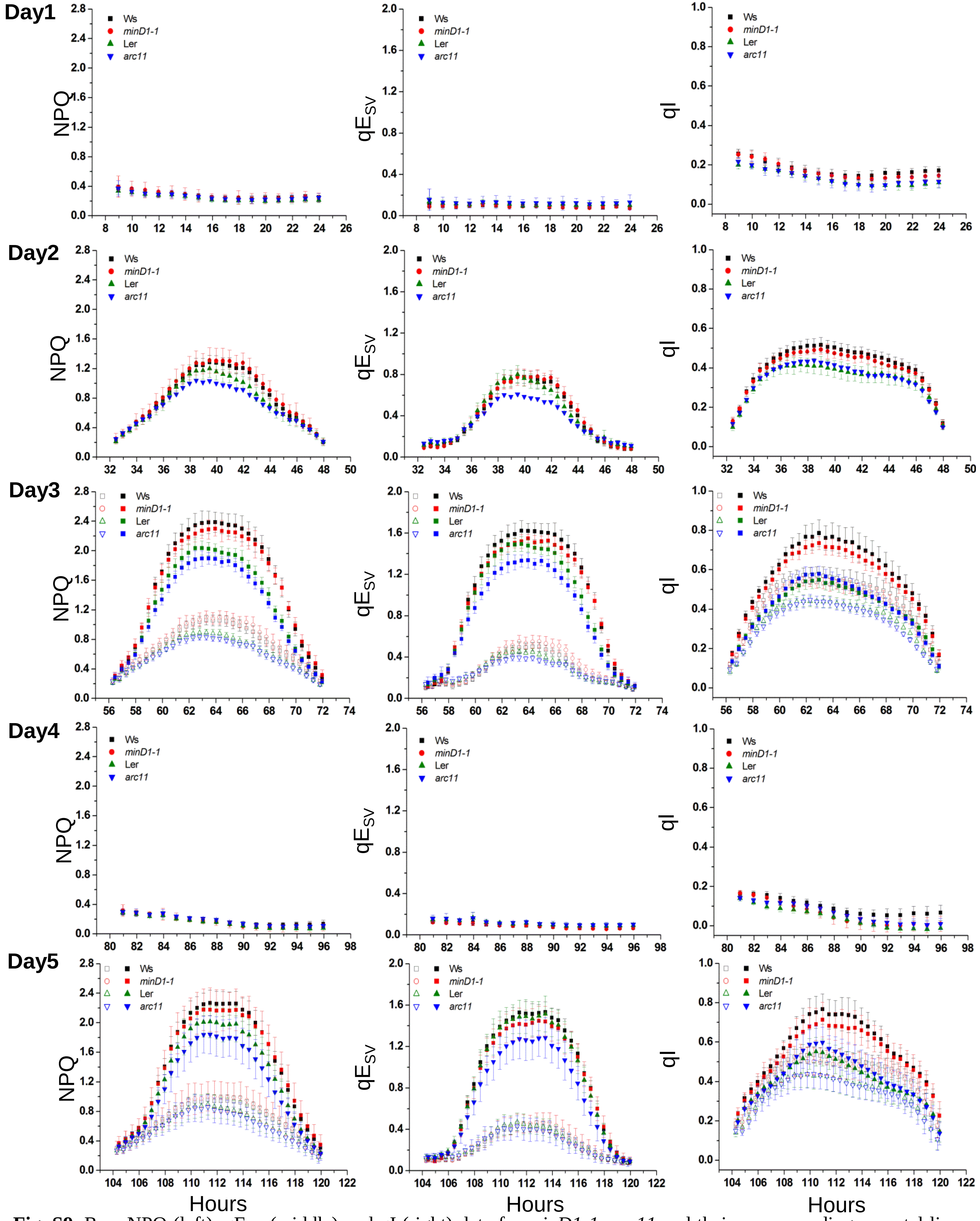

Fig. S9. Raw NPQ (left), qEsv (middle) and qI (right) data for *minD1-1*, *arc11* and their corresponding parental lines used to generate results shown in Figure 4. The open and closed symbols on Days 3 and 5 correspond to alternating periods of ambient and fluctuating light, respectively. Each data point represents the mean of 6-10 plants; error bars represent SD.

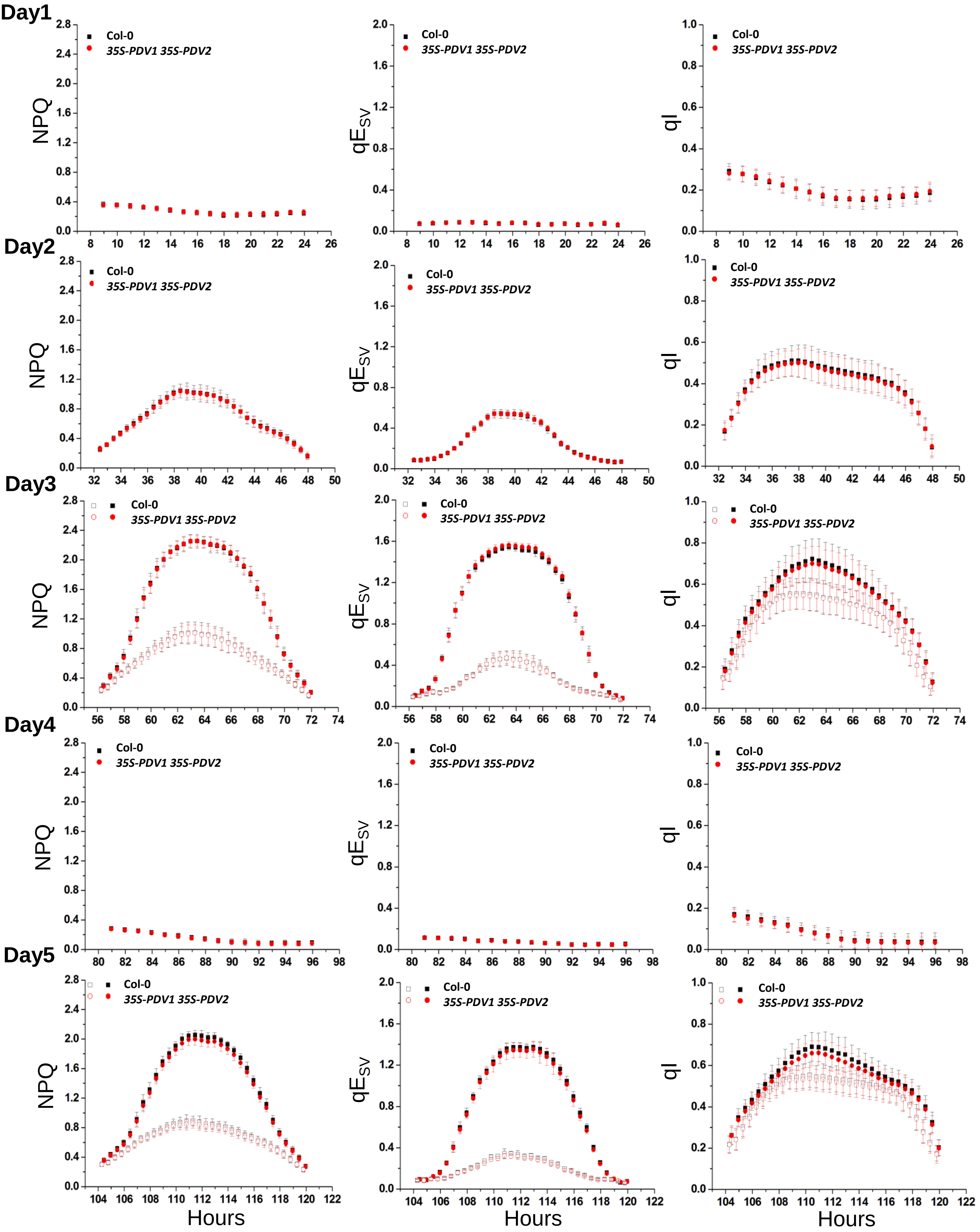

Fig. S10. Raw NPQ (left panel), qEsv (middle panel) and qI (right panel) for *35S-PDV1 35S-PDV-2* and corresponding wild type (Col-0) shown in Figure 4. The open and closed symbols on Days 3 and 5 correspond to alternating periods of ambient and fluctuating light, respectively. Each data point represents the mean of 6-10 plants; error bars represent SD.

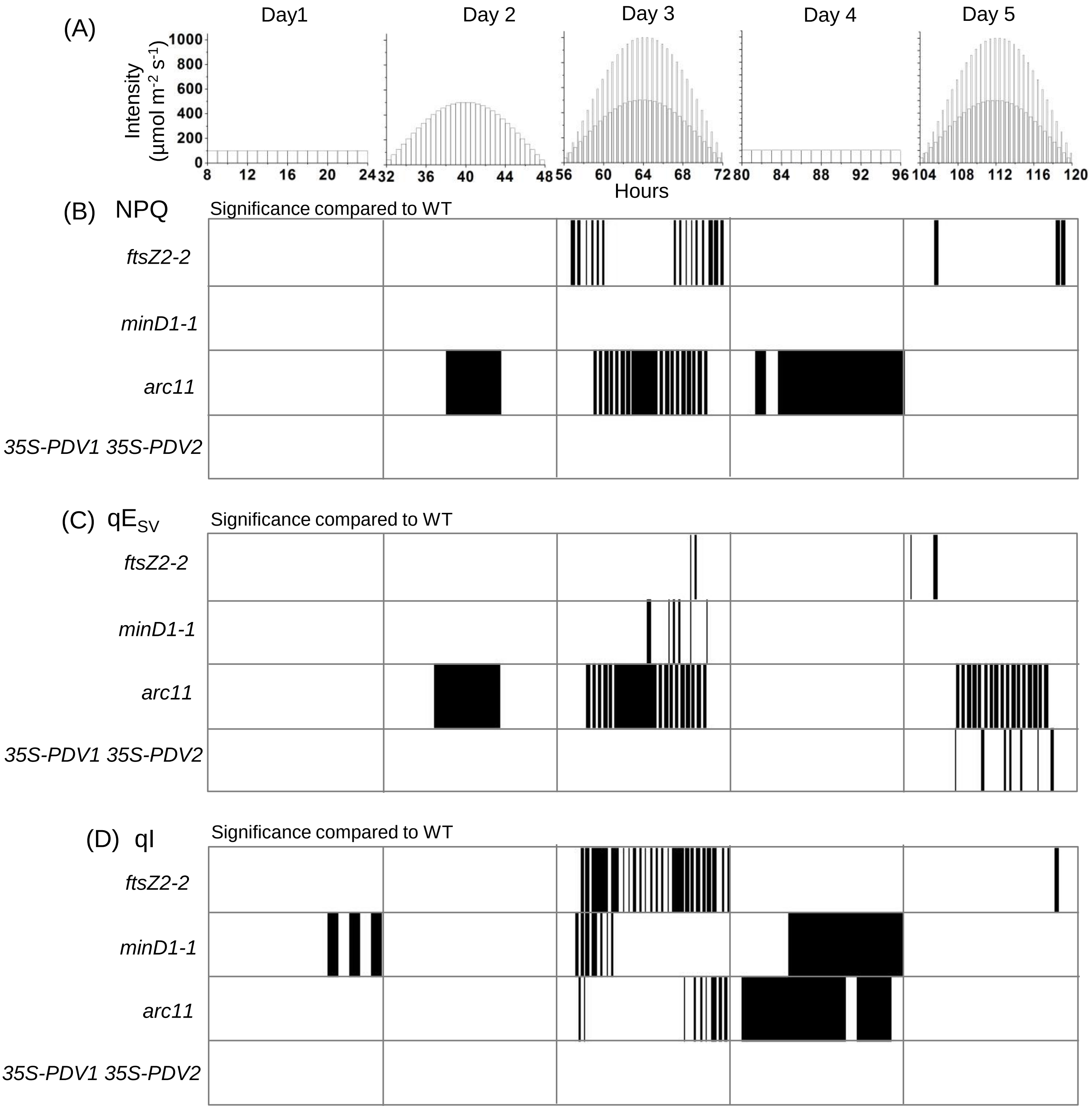

Fig. S11. Heat maps showing the statistical significance of differences in photosynthetic parameters at each time point for data shown in Figure 4. (A) Light regime. (B-D) Statistical significance of differences in in NPQ (B), qE_{SV} (C), and qI (D) values between WT and the indicated genotypes at each measurement time point. Differences were evaluated using the unpaired, two-tailed t-test. P values ≤ 0.05 were taken as statistically significant (black stripes).

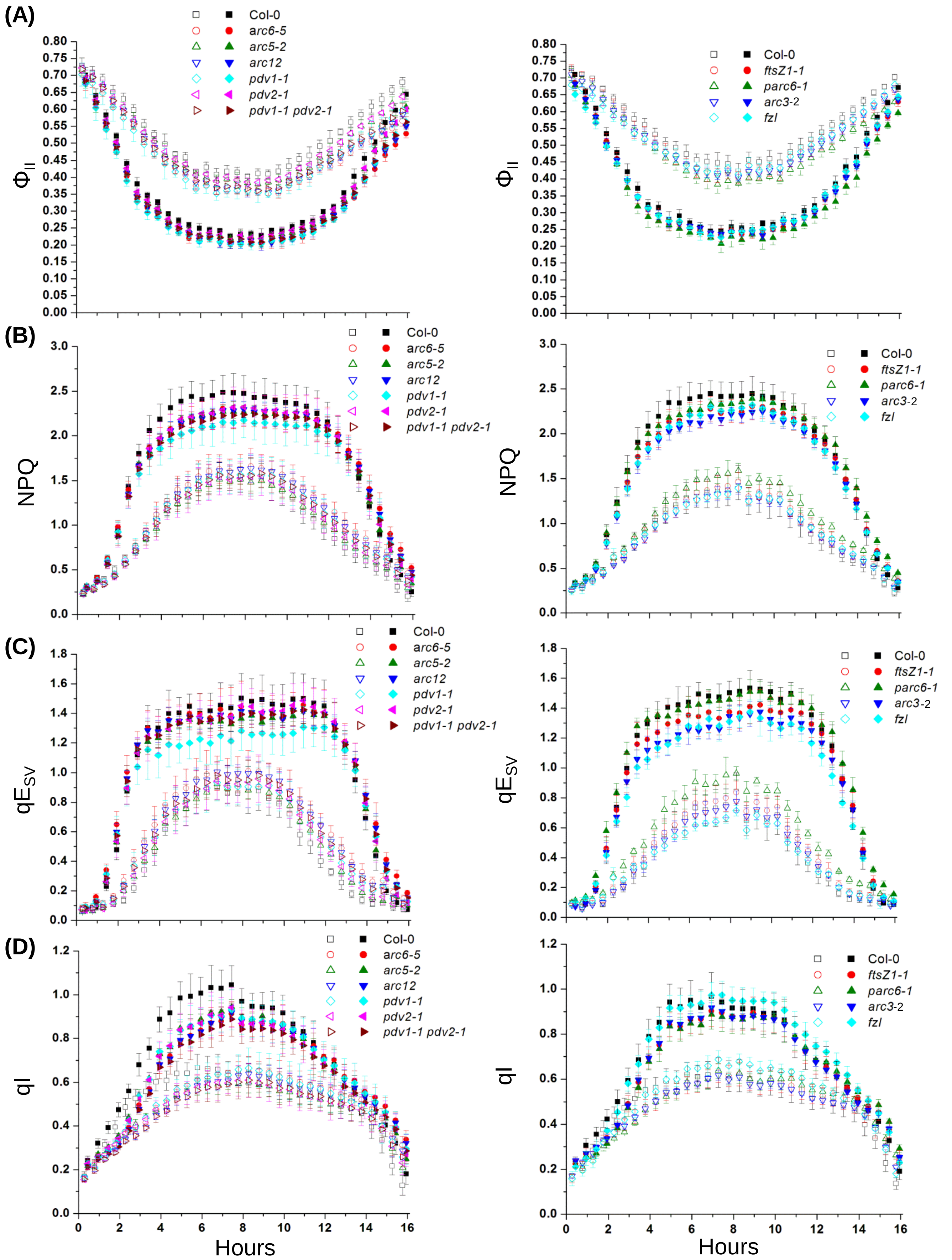

Fig. S12. Raw data for (A) Φ_{II} and "apparent" (traditional) (B) NPQ, (C) qE_{SV} and (D) qI values for the experiment shown in Figure 5B and 6A-C, but uncorrected for chloroplast movements. The open and closed symbols correspond to alternating periods of ambient and fluctuating light, respectively. For all data points, n = 4-6 and error bars represent SD. The data for large and intermediate chloroplast mutants are shown in the left and right panels, respectively.

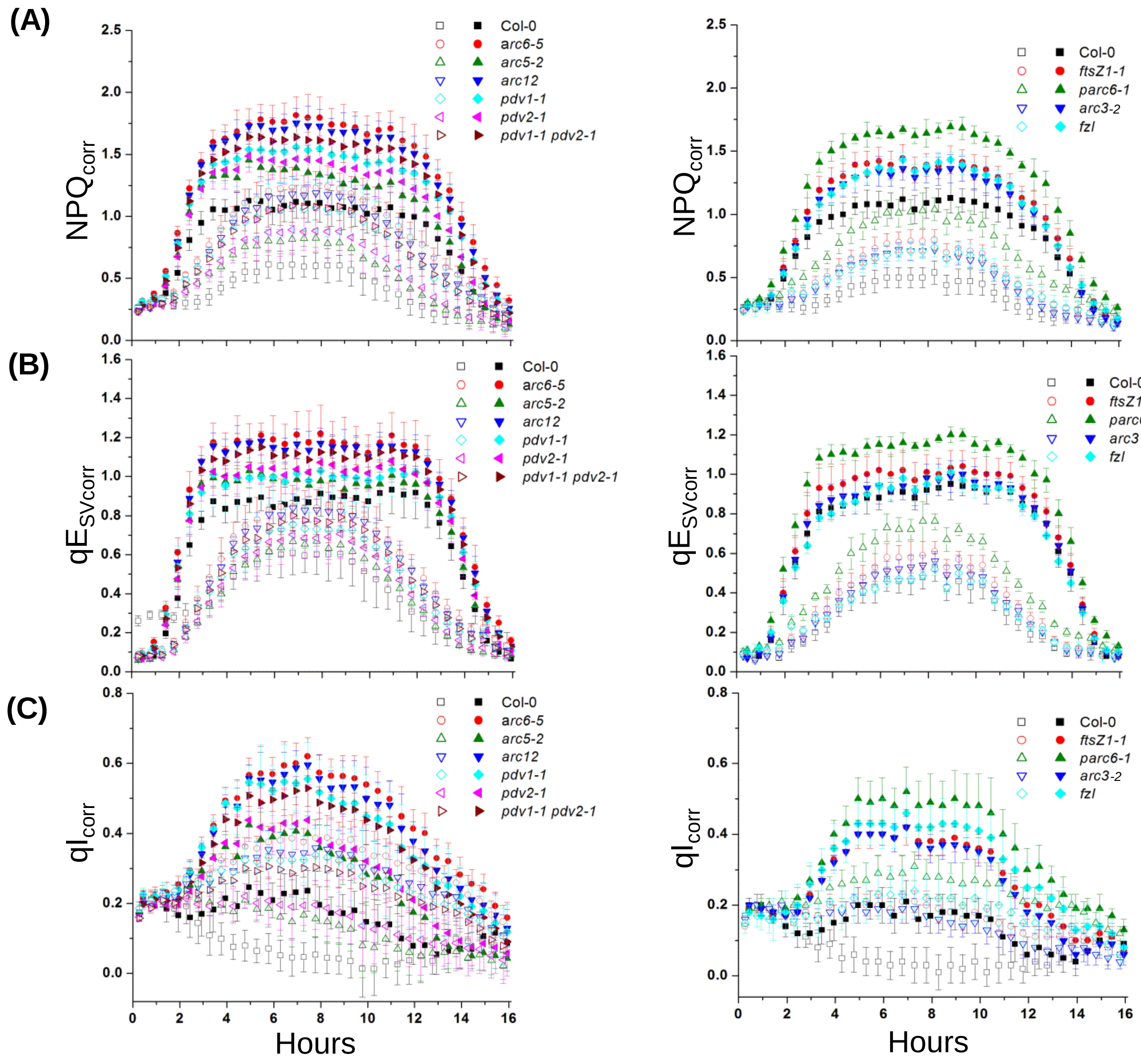

Fig. S13. Raw NPQ_{corr} (A), qE_{SVcorr} (B) and qI_{corr} (C) data for results shown in Figure 6A-C. The open and closed symbols correspond to alternating periods of ambient and fluctuating light, respectively. For all data points, n = 4-6 and error bars represent SD. The data for large and intermediate chloroplast mutants are shown in the left and right panels, respectively.

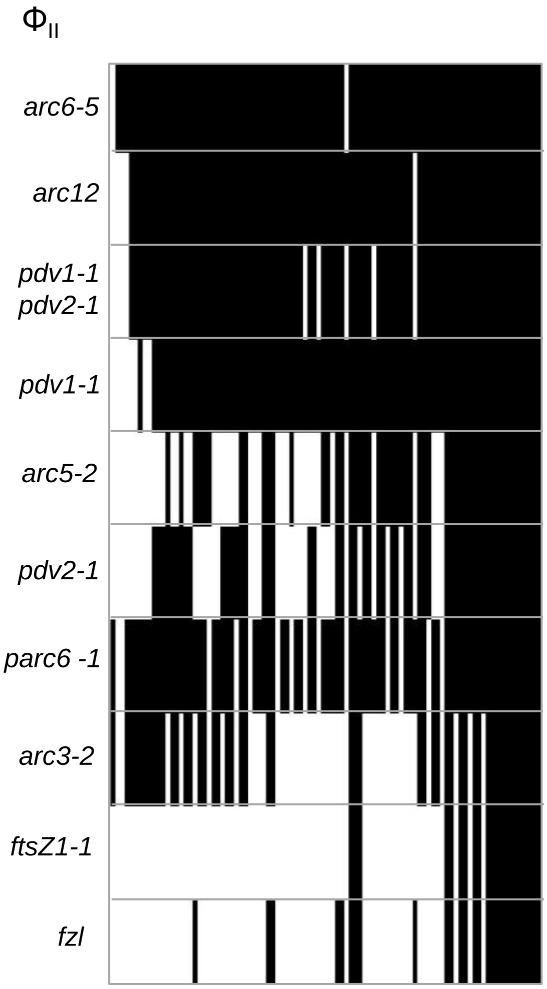

Fig. S14. Heat map showing the statistical significance of differences in Φ_{II} between WT and indicated genotypes at each measurement time point for the data shown in Figures 5B and S12A. Differences were evaluated using the unpaired, two-tailed t-test. P values ≤ 0.05 were taken as statistically significant (black stripes).

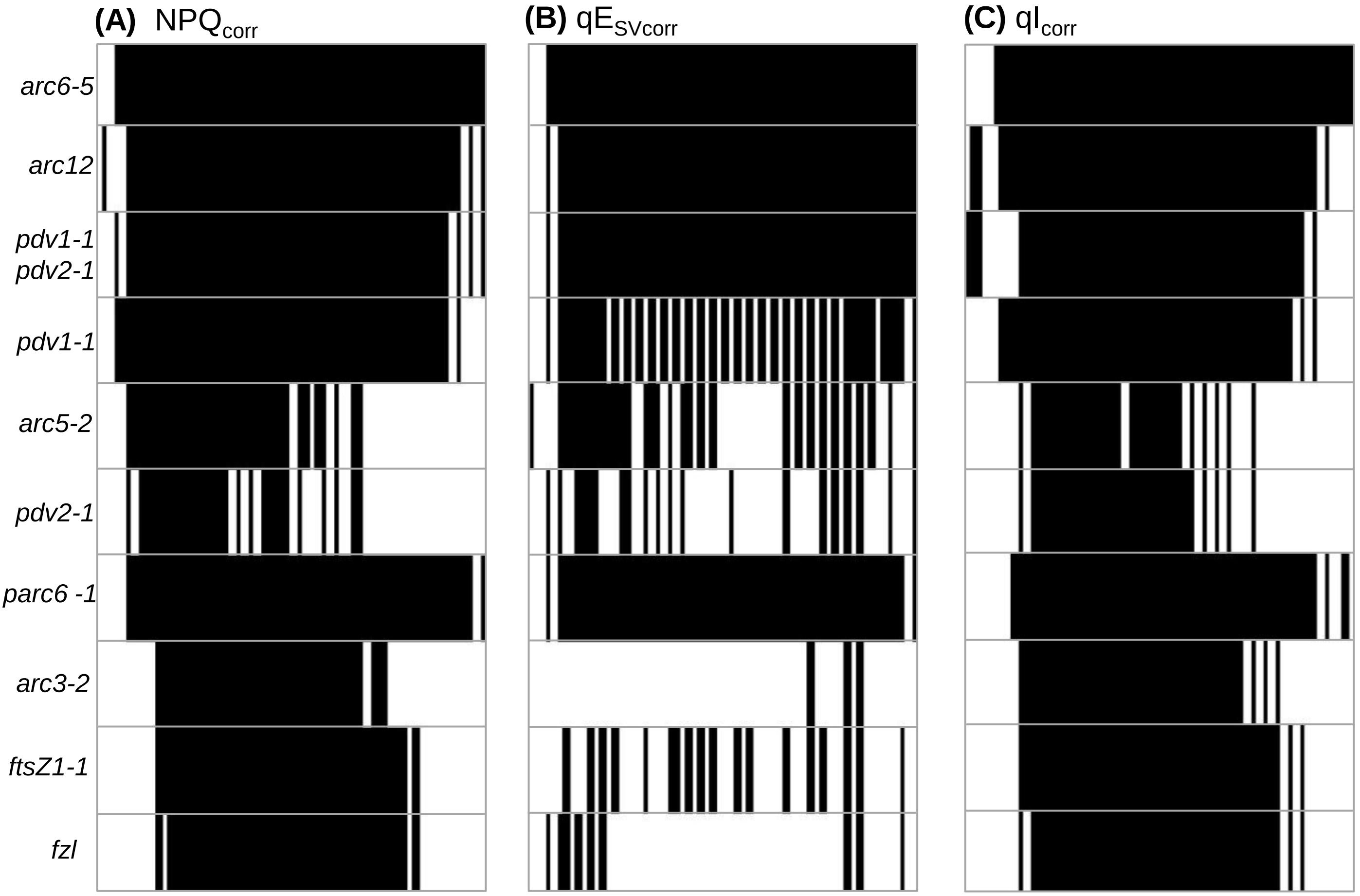

Fig. S15. Heat maps showing the statistical significance of differences in photosynthetic parameters at each time point for data shown in Figure 6. (A) NPQ_{corr}, (B) qE_{SVcorr} and (C) qI_{corr} values in the indicated genotypes relative to those in WT. Differences were evaluated using the unpaired, two-tailed t-test. P values ≤ 0.05 were taken as statistically significant (black stripes).
